# Peptide hormones in shaping root system architecture and environmental adaptation: Current advances and translational perspectives

**DOI:** 10.1016/j.xplc.2026.101987

**Published:** 2026-07-02

**Authors:** Xuechen Zhao, Hongqin Tan, Gaojian Li, Yang Xu, Zhaojun Ding, Zipeng Yu

**Affiliations:** 1 The Key Laboratory of Plant Development and Environmental Adaptation Biology, Ministry of Education, Shandong Key Laboratory of Precision Molecular Crop Design and Breeding, Shandong Provincial Key Laboratory of Plant Stress Biology and Genetic Improvement, School of Life Sciences, Shandong University, Qingdao 266237, China; 2 College of Life Sciences, Shaanxi Normal University, Xi’an 710119, China; 3 Shandong Peanut Research Institute, Shandong Academy of Agricultural Sciences, Qingdao 266100, China

**Keywords:** root system architecture, RSA, peptide hormones, RSA plasticity, crop development, crop environmental adaptation

## Abstract

Root system architecture (RSA) is pivotal to plant nutrient acquisition and environmental adaptation. In recent years, peptide hormones—intercellular signaling molecules that act locally or systemically—have emerged as critical regulators of RSA. These hormones are recognized by specific receptor kinases, which transduce peptide signals by activating downstream pathways involving calcium fluxes, reactive oxygen species bursts, and mitogen-activated protein kinase cascades, or by directly modulating core signaling components. These signaling networks integrate endogenous developmental cues with exogenous environmental stimuli to fine-tune root growth and development, thereby shaping RSA plasticity. This review provides a systematic analysis of the essential roles of peptide hormones in regulating RSA plasticity, elucidates their associated molecular pathways, and critically assesses their potential to optimize crop RSA, improve nutrient use efficiency, and enhance stress resistance, thereby contributing to sustainable agriculture.

## Teaser

Peptide hormones are key signaling molecules that regulate plant root system architecture (RSA) by integrating developmental and environmental cues through receptor kinase-mediated signaling networks. This review summarizes the regulatory mechanisms by which peptide hormone signaling controls RSA plasticity during primary root growth, lateral root formation, root hair development, and nodulation. It also highlights how these peptide–receptor modules represent promising targets for improving crop root architecture, nutrient use efficiency, and stress resilience.

## Introduction

The emergence of roots not only improved plant anchorage but also significantly enhanced water and mineral nutrient uptake, thereby supporting the adaptation of plants to terrestrial environments (Kenrick and Strullu-Derrien, 2014; Fang et al., 2021a). During subsequent evolution, plant root systems became increasingly complex, giving rise to highly specialized structures such as primary roots, lateral roots (LRs), root hairs, and adventitious roots (Sharma et al., 2021). In legumes, nitrogen acquisition was further enhanced by the evolution of root nodules, which form through symbiotic associations with rhizobia (Cervantes-Perez et al., 2024). Collectively, these structures constitute the root system architecture (RSA), which directly influences plant growth, nutrient acquisition efficiency, and stress tolerance (Karlova et al., 2021; Zhang et al., 2025c).

To dynamically adjust RSA in response to variable soil environments, plants integrate hormonal cues, including auxin and cytokinin (CK), with key developmental regulators that fine-tune root cell fate (Roychoudhry and Kepinski, 2022). In addition to classical phytohormones, secreted peptide hormones have emerged as important regulators of RSA (Keerthana et al., 2025). Unlike traditional plant hormones, which are classified by chemical properties, secreted small peptides are low-abundance but highly versatile signaling molecules. After undergoing short- or long-distance transport, they are recognized by plasma membrane (PM)-localized receptor kinases, thereby triggering intracellular signaling cascades (Zhang et al., 2025d). Similar to classical plant hormones, peptide hormones regulate embryonic development, organogenesis, and responses to pests and diseases (Yu et al., 2020b; Zhang et al., 2025d). Importantly, they also shape RSA by coordinating developmental programs with environmental cues.

Numerous peptide hormones have been demonstrated to play crucial roles in shaping RSA, including but not limited to phytosulfokine (PSK), casparian strip integrity factor (CIF), clavata3 (CLV3)/embryo surrounding region-related (CLE) peptides, C-terminally encoded peptide (CEP), rapid alkalinization factor (RALF), root growth factor (RGF; also known as golven, GLV), and inflorescence deficient in abscission (IDA) (Keerthana et al., 2025; Zhang et al., 2025d). These peptide hormones regulate RSA through diverse mechanisms: some act directly on root developmental programs, whereas others influence RSA by modulating classical phytohormone pathways, either directly or indirectly (Li et al., 2025). Here, we systematically review the regulatory mechanisms of peptide hormones in plant RSA, focusing on their critical roles in primary root growth, LR formation, root hair development, nodulation, and root regeneration. We begin by examining the molecular mechanisms elucidated in *Arabidopsis* and then discuss how these insights may be translated to improve RSA in staple and oilseed crops. Finally, we discuss the potential applications of peptide hormone signaling in future agricultural innovation.

## Overview of peptide hormones

Small peptides can be broadly classified into secreted and non-secreted types. This review focuses primarily on secreted peptides, also referred to as peptide hormones, which act as extracellular signaling molecules that mediate intercellular communication (Zhang et al., 2025d). Peptide hormones are generally defined as short polypeptides of fewer than 100 amino acids and can be classified into three main categories: cysteine-rich peptides, small linear peptides that undergo post translational modifications (PTMs), and non-cysteine-rich/non-PTM peptides (Xiao et al., 2025). Cysteine-rich peptides possess multiple conserved and typically even-numbered cysteine residues that form disulfide bonds, resulting in compact folded structures that are thermostable and resistant to proteolysis (Ma et al., 2023). Small linear peptides often carry PTMs such as tyrosine sulfation, proline hydroxylation, or hydroxyproline arabosylation (Chekan et al., 2024; Li et al., 2025).

### Processing of peptide hormones

**Figure 1:**
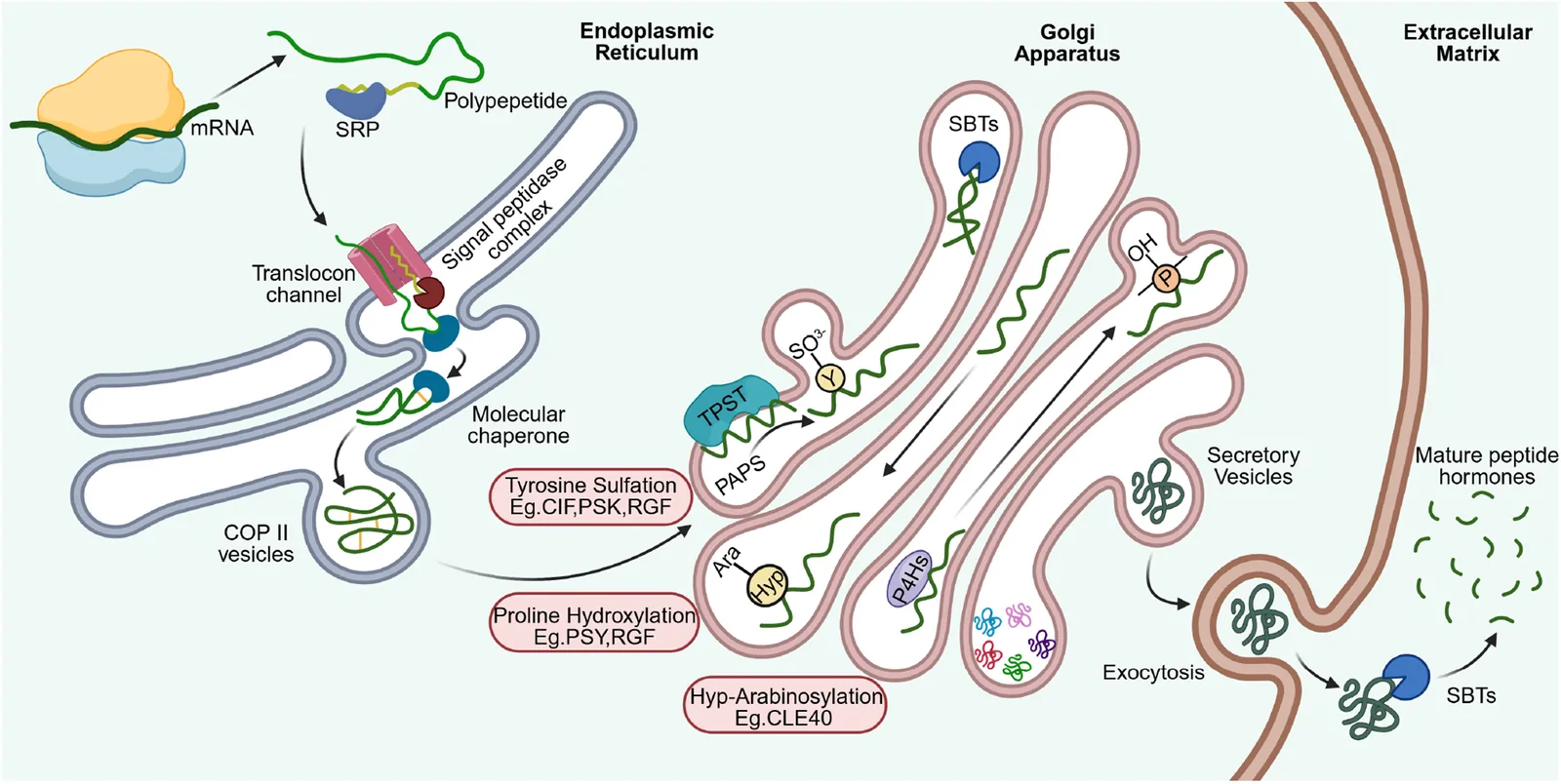
Pathway of peptide hormone processing and maturation. Peptide precursors are synthesized on ribosomes, and their N-terminal signal peptides are recognized by the cytosolic SRP. The ribosome–nascent peptide–SRP complex is targeted to and engages the endoplasmic reticulum (ER) membrane translocon, through which the polypeptide enters the ER lumen. The signal peptide is cleaved by signal peptidase, while molecular chaperones facilitate protein folding and disulfide bond formation. Subsequently, the peptide precursors are transported to the Golgi apparatus via COPII vesicles, where they undergo diverse PTMs. TPST catalyzes tyrosine sulfation, thereby enhancing peptide–receptor binding affinity; P4Hs mediate proline hydroxylation, and the subsequent arabinosylation of hydroxyproline further enhances peptide stability and receptor recognition. The modified precursors are packaged into secretory vesicles and released into the apoplast through exocytosis at the plasma membrane (PM). SBT proteases then remove flanking or inhibitory sequences to generate mature peptides. This ordered, multi-organelle processing ensures the precise spatiotemporal control of peptide maturation and thereby regulates plant root developmental signaling. SRP, signal recognition particle; Hyp-Ara, hydroxyproline arabinosylation; PAPS, 3′-phosphoadenosine-5′-phosphosulfate; P4Hs, prolyl 4-hydroxylases; SBT, subtilisin-like protease; TPST, tyrosylprotein sulfotransferase.

Most peptide hormones are translated as precursor proteins that contain an N-terminal secretion signal peptide approximately 20–30 amino acids in length (Figure 1). During translation, the signal peptide is recognized by the cytosolic signal recognition particle, which directs the ribosome–nascent peptide complex to the translocon in the endoplasmic reticulum (ER) membrane. The nascent polypeptide is then cotranslationally translocated across the membrane into the ER lumen. As translocation proceeds, the signal peptidase complex in the ER membrane recognizes the cleavage site between the signal peptide and the propeptide and hydrolytically removes the signal peptide. Concurrently, molecular chaperones within the ER lumen facilitate the initial folding of the peptide chain and, where applicable, the formation of stable disulfide bonds, thereby generating a properly folded precursor (Matsubayashi, 2018; Luxmi and King, 2024; Zhang et al., 2025d).

Subsequently, these precursors are transported from the ER to the Golgi apparatus via COPII-coated vesicles. Within the Golgi, they undergo additional PTMs, ultimately yielding biologically active secreted peptide signals (Luxmi et al., 2022). On the luminal surface of the Golgi apparatus, tyrosyl protein sulfotransferase (TPST) utilizes 3′-phosphoadenosine 5′-phosphosulfate as a sulfate donor to catalyze the sulfation of specific tyrosine residues. This modification substantially enhances the binding affinity of peptides such as CIF, PSK, and RGF to their receptors (Zhang et al., 2025d), highlighting the importance of tyrosine sulfation for peptide recognition. The *tpst-1* mutant exhibits pronounced root development defects, including reduced root meristem size and impaired coordination of cell elongation and expansion (Kaufmann et al., 2021). Moreover, different tyrosine-sulfated peptides, such as PSK, PSY1, and RGF1, exhibit complementary roles in rescuing the root defects of *tpst-1*: PSK and PSY1 restore cell elongation in the elongation zone, whereas RGF1 rescues the meristem size defect (Kaufmann et al., 2021). Together, these findings indicate that TPST-mediated sulfation of multiple peptide hormones plays a pivotal role in root development.

In addition to sulfation, proline hydroxylation, catalyzed by prolyl 4-hydroxylases, represents another crucial PTM that occurs in the Golgi apparatus. For example, hydroxylation of the fourth proline residue in the CLE40 precursor prevents secondary cleavage by subtilisin-like proteases (SBTs), thereby promoting the production of active peptides. SBTs are serine proteases that process a wide range of plant proteins. In addition to regulating CLE40 maturation, they cleave diverse peptide hormone precursors to remove inactive N- and/or C-terminal regions and generate mature, bioactive peptides (Stührwohldt et al., 2020). Hydroxyproline arabinosylation is another PTM that can occur in the Golgi apparatus. This modification is commonly observed in peptides such as PSY and RGF and can increase peptide stability and promote effective receptor recognition (Shinohara and Matsubayashi, 2013).

After processing and modification within the Golgi apparatus, peptide precursors are packaged into Golgi-derived secretory vesicles and delivered to the PM (Stührwohldt et al., 2020). Upon fusion with the PM, these vesicles release their contents into the apoplast. The spatiotemporal specificity of peptide production and release is governed by multiple layers of control: (1) cell-type-specific expression of peptide precursors and their processing enzymes, including SBTs, restricts active peptide production to particular cell types and developmental stages; (2) subcellular trafficking and polarized secretion direct secretory vesicles to specific PM domains; and (3) post-translational modifications influence processing efficiency, secretory competence, and peptide activity. Ultimately, under precise spatiotemporal control, SBTs catalyze the final proteolytic of these precursors, removing propeptide sequences to release mature, active peptides (Figure 1). Through this coordinated interplay between organelles and enzymatic cascades, peptide signaling molecules achieve spatiotemporal specificity in their production, modification, and release, thereby ensuring the precise execution of their biological functions.

### Recognition mechanisms of peptide hormones

As crucial signaling molecules, peptide hormones convey developmental and stress-related information from signal-emitting cells to signal-receiving cells via short- or long-distance transport (Zhang et al., 2024b). At target cells, peptide ligands are perceived by cognate receptors localized at the PM. These receptors typically belong to the leucine-rich repeat receptor-like kinase (LRR-RLK) family (Zhang et al., 2024b). A typical LRR-RLK comprises three regions: an extracellular LRR-containing ligand-binding domain, also known as the receptor domain; a single transmembrane helix; and an intracellular kinase domain (Zhang et al., 2024b).

Some receptors require co-receptors because their extracellular domains alone cannot form a fully competent ligand-binding or signaling complex (Wu et al., 2024). However, even canonical typical LRR-RLKs require co-receptors for efficient ligand recognition and signal activation. For instance, the extracellular domains of the receptor and co-receptor may jointly engage the ligand, effectively enclosing it within the receptor complex (Soltabayeva et al., 2022). The co-receptor repertoire is relatively limited and consists primarily of members of the somatic embryogenesis receptor-like kinase (SERK) family, which comprises five members: SERK1, SERK2, SERK3 (also known as BAK1), SERK4 (also known as BKK1), and SERK5. These co-receptors can heterodimerize with diverse ligand-binding receptor kinases, thereby stabilizing receptor complexes and facilitating reciprocal phosphorylation between the intracellular kinase domains to activate downstream signaling (Fan et al., 2016). For example, during RGF1 recognition, RGF1 binds via its sulfated RxGG motif to the extracellular LRR domains of its cognate RGF1-insensitive (RGI) receptors, thereby recruiting SERK-family co-receptors to form an active receptor complex (Yang et al., 2020b; Yamada et al., 2020). Similarly, during PSK recognition, binding of PSK to phytosulfokine receptor 1 (PSKR1) promotes association with BAK1, which contributes to receptor activation and downstream signal transduction (Yu et al., 2020a; Kaufmann et al., 2021; Li et al., 2023). Genetic evidence suggests that loss of SERK function significantly impairs root responses to multiple peptides, including RGF and PSK, highlighting the broad importance of SERKs in peptide-mediated signaling (Ou et al., 2022). However, how these peptide signaling modules interact genetically to regulate RSA—that is, their epistatic relationships—remains largely unexplored.

In addition to SERKs, other receptor-like kinases and receptor-like proteins contribute to ligand-specific receptor complexes. For example, during CLE40 recognition, *Arabidopsis* crinkly4 (ACR4), clavata1 (CLV1), and CLV3 insensitive receptor kinases (CIKs) function together in receptor complexes that mediate CLE40 perception and signaling (Li et al., 2025). In signaling mediated by CLE17 and CLE19, receptor-like protein kinase 2 (RPK2) and the CLV2–coryne (CLV2–CRN) complex serve as major signaling components (Jeon et al., 2021). These diverse receptor–co-receptor assemblies illustrate how plants combine shared and ligand-specific signaling components to distinguish among peptide hormones and coordinate diverse aspects of root development (Table 1).

**Table 1:** Peptide hormones involved in the regulation of plant root system architecture (RSA).

Subfamily	Species	Peptide hormone	Receptor	Function	Evidence	References
RALF	*Arabidopsis thaliana*	AtRALF1	AtFERONIA	suppressing primary root elongation	*fer-4* roots are insensitive to RALF1	Du et al. (2016)
				promoting polarized root hair growth	*fer-4* roots are insensitive to RALF1-induced polarized root hair growth	Zhu et al. (2020b)
	*Fusarium oxysporum*	F-RALF	AtFERONIA	inhibiting root hair elongation	exogenous application of F-RALF	Masachis et al. (2016)
	*Arabidopsis thaliana*	AtRALF34	AtTHE1	suppressing LR development	*the1* mutants exhibit increased LR density	Li et al. (2025)
	*Arabidopsis thaliana*	AtRALF33	AtFERONIA	initiating root regeneration	*fer* mutants exhibit an enhanced regeneration capacity	Shen et al. (2025)
	*Oryza sativa*	OsRALF34	unknown	promoting LR development	overexpression of OsRALF34	Roy et al. (2024)
CIF	*Arabidopsis thaliana*	AtCIF1/AtCIF2	GSO1/SGN3	regulating Casparian strip formation	*serk3* mutants exhibit delayed Casparian strip formation	Jeon et al. (2021)
	*Oryza sativa*	OsCIF1/OsCIF2	OsSGN3a/OsSGN3b	influencing the spatial distribution of LRPs	*cif1 cif2* double mutants exhibit reduced LR density	Roy et al. (2024)
CLE	*Arabidopsis thaliana*	AtCLE40	ACR4	promoting QC activity	exogenous application of CLE40p	Berckmans et al. (2020)
	*Arabidopsis thaliana*	AtCLE25/26/45	BAM1/BAM3	sustaining normal primary root development	the *cle25;26;45* triple mutant rescues the phenotypes of *brx* and *ops* mutants	Hu et al. (2022)
	*Arabidopsis thaliana*	AtCLE8	unknown	regulating embryo and endosperm development	the *cle8-1* mutant exhibits aberrant endosperm development	Fiume and Fletcher (2012)
	*Arabidopsis thaliana*	AtCLE41	PXY	promoting primary root growth, guiding LRP initiation, and promoting regeneration	exogenous application of TDIF	Yang et al. (2020a); Hirakawa et al. (2010)
	*Arabidopsis thaliana*	AtCLE14	CLV2/PEPR2	inducing root growth arrest	overexpression of *CLE14*	Gutierrez-Alanis et al. (2017)
				increasing root hair number	exogenous application of CLE14	Zhou et al. (2025)
	*Arabidopsis thaliana*	AtCLE17	RPK2	controlling cell proliferation in the RAM	*rpk1* and *rpk2* mutants exhibit abnormal PIN1 localization and reduced DR5 signal	Jeon et al. (2021)
	*Arabidopsis thaliana*	AtCLE19/AtCLE27	PXL1/unknown	reducing LR formation	exogenous application of CLE19/CLE27	Fiers et al. (2005); Czyzewicz et al. (2015)
	*Oryza sativa*	OsCLE402	unknown	terminating RAM activity	exogenous application of FCP2p	Chu et al. (2013)
	*Triticum aestivum*	TaCLE3	unknown	reducing primary root length	exogenous application of TaCLE3p	Li et al. (2019)
	*Triticum aestivum*	TaCLE24b	TaCLV1	promoting LRP formation	exogenous application of TaCLE24b	Yang et al. (2025)
	*Lotus japonicus*	LjCLE-RS1/LjCLE-RS2	HAR1/KLV	suppressing subsequent nodule initiation and development	overexpression of *LjCLE-RS1* or *LjCLE-RS2*	Yamada and Sawa (2013); Okamoto et al. (2013)
	*Medicago truncatula*	MtCLE12/MtCLE13	SUNN		overexpression of *MtCLE12* or *MtCLE13*	
	*Medicago truncatula*	MtCLE33	SUNN	suppressing nodule formation	overexpression of *MtCLE33*	Muller et al. (2019)
CEP	*Arabidopsis thaliana*	AtCEP5	AtCEPR1	suppressing LR growth	exogenous application of CEP5	Chapman et al. (2019)
	*Arabidopsis thaliana*	AtCEP1	AtCEPR1	repressing root elongation and mediating nitrogen-deprivation signaling between LRs	overexpression of *AtCEP1*	Taleski et al. (2023)
	*Triticum aestivum*	TaCEP1	TaCEPRL	repressing primary root elongation	overexpression of *TaCEP1*	Wen Yang et al. (2025)
	*Medicago truncatula*	MtCEPs	MtCRA2	promoting nodule formation	*cra2* mutants are insensitive to MtCEP-induced promotion of nodulation	Laffont et al. (2019)
	*Medicago truncatula*	MtCEP1	MtCRA2	inhibiting LR formation	*cra2* mutants are insensitive to MtCEP1-induced LR inhibition	Taleski et al. (2018)
	*Oryza sativa*	OsCEP6.1	unknown	inhibiting LR development	exogenous application of OsCEP6.1	Sui et al. (2016)
PSK	*Arabidopsis thaliana*	AtPSK	AtPSKR1/AtPSKR2	promoting cell-wall loosening and rapid cell expansion and influencing atrichoblast fate	database analysis; increased expression of *WER*, *MYB23*, and *At1g66800* in *tpst-1*	Kaufmann et al. (2021)
	*Triticum aestivum*	TaPSK5	PSKR	promoting root elongation	overexpression of *TaPSK5-D* and application of *rTaPSK5-D*	Geng et al. (2020)
	*Cunninghamia lanceolata*	ClPSK1/ClPSK2	PSKR	upregulating the expression of genes involved in root morphogenesis	overexpression of *ClPSK1* or *ClPSK2*	Wu et al. (2019)
	*Medicago truncatula*	MtPSK-ε	MtPSKR	increasing LR number	overexpression of *MtPSK-ε*	Di et al. (2022)
	*Medicago truncatula*	MtPSK-δ	MtPSKR	facilitating nodule formation	overexpression of *MtPSK-*δ	Yu et al. (2022a)
miPEP	*Arabidopsis thaliana*	AtmiPEP165a	unknown	increasing primary root length and suppressing LR formation	exogenous application of miPEP165a	Ormancey et al. (2020)
	*Medicago truncatula*	MtmiPEP171b	unknown	reducing LR formation	exogenous application of miPEP171b	Lauressergues et al. (2015)
PEP	*Oryza sativa*	OsPEP1	unknown	suppressing cell division in the meristem zone	exogenous application of OsPEP1	Xiang et al. (2021)
	*Solanum lycopersicum*	SlREF1	SlPORK1	initiating cell reprogramming	exogenous application of REF1p	Ikeuchi et al. (2019)
SCOOP	*Arabidopsis thaliana*	AtSCOOP12	AtMIK2	influencing root growth	analysis of transgenic plants expressing MIK2	Hou et al. (2021)
RGF	*Arabidopsis thaliana*	AtRGF1	AtRGI	promoting RAM enlargement, influencing normal root hair development, and reconstructing the regenerative stem-cell system	exogenous application of RGF1	Yamada et al. (2020); Wang et al. (2025a)
	*Arabidopsis thaliana*	AtGLV6/10	AtRGI	inhibiting LR formation	overexpression or exogenous application of *GLV6/GLV10*	Fernandez et al. (2020)
	*Prunus persica*	pCTG134	unknown	promoting LR development	overexpression or exogenous application of *pCTG134*	Busatto et al. (2017)
	*Zea mays*	ZmRGF1	ZmRGI	promoting RAM enlargement	overexpression of *ZmRGF1*	Fang et al. (2021b)
	*Solanum lycopersicum*	SlRGF1	SlRGI	altering gravitropic responses	overexpression of *SlRGI*	Fang et al. (2021b)
	*Medicago truncatula*	MtGLVs	MtRGI	reducing nodule number	overexpression or exogenous application of MtGLVs	Roy et al. (2024)
IDA	*Arabidopsis thaliana*	AtIDA	AtHAE/AtHSL2	facilitating lateral root emergence	*hae hsl2* double mutants exhibit altered LRP morphogenesis	Zhu et al. (2019)
PLS	*Arabidopsis thaliana*	AtPLS	AtETR1	mediating root cell elongation	*pls* mutants exhibit short roots	Chilley et al. (2006)
SiDVL	*Setaria italica*	SiDVL	unknown	inhibiting root elongation	overexpression of *SiDVLs*	Wang et al. (2024)
PSY	*Arabidopsis thaliana*	AtPSY1	AtPSY1R	enhancing LRP formation	*psy1r* mutants exhibit reduced primary root elongation and LR density	Tost et al. (2021)
	*Zea mays*	ZmPSY3	unknown	regulating root carotenoid and ABA biosynthesis under abiotic stress	quantitative RT–PCR analysis	Li et al. (2008)
LOHN	*Arabidopsis thaliana*	AtLOHN1	unknown	regulating LR spacing	*lohn1* mutants exhibit increased LR density; exogenous application of LOHN1 restores wider LR spacing	Ito et al. (2025)
DVL	*Arabidopsis thaliana*	AtDVL	unknown	maintaining root-tip cell viability and proliferative capacity	multiple *dvl* mutants exhibit altered root phenotypes	Yang et al. (2024a)
IMA	*Lotus japonicus*	LjIMA	unknown	promoting nodule formation	*ima* mutants exhibit reduced root nodulation	Ito et al. (2024)
	*Arabidopsis thaliana*	AtIMA	unknown	regulating nitrogen homeostasis	*ima* mutants exhibit abnormal growth and altered nitrogen metabolism	Ito et al. (2024)

The specificity of ligand recognition is determined by multiple layers of molecular control. PTMs, such as sulfation of the two tyrosine residues in PSK, create distinctive structural determinants essential for receptor binding (Li et al., 2023). The architecture of the receptor ectodomain provides an additional layer of specificity. For example, the LRR domain of CLV1 contributes directly to the formation of its ligand-binding pockets (Shinohara and Matsubayashi, 2013).

### Intracellular signaling cascades of peptide hormones

Upon perception of peptide hormones, target cells initiate a series of highly coordinated intracellular signaling events. These events include the generation of second messengers, the activation of protein phosphorylation cascades and specific transcription factors, and alterations in cell wall properties. Together, these integrated responses direct cellular developmental programs and thereby shape overall RSA in response to environmental and physiological cues (Furumizu and Aalen, 2023; Zhang et al., 2025d).

Second messengers, especially calcium ions (Ca^2+^), play a crucial role in amplifying peptide-derived signals. Upon ligand perception, activated receptors rapidly induce transient increases in cytoplasmic Ca^2+^ concentrations. These Ca^2+^ signals are further transduced by downstream components, such as calcium-dependent protein kinases (CDPKs or CPKs), thereby influencing processes including cell division and differentiation (Yamada et al., 2020; Yang et al., 2023; Zhang et al., 2025a). Transient bursts of reactive oxygen species (ROS), followed by the re-establishment of redox homeostasis, also constitute a common signaling response. Certain peptide hormones regulate NADPH oxidase activity, leading to localized ROS accumulation. ROS not only function directly as signaling molecules affecting protein function and stability but also participate in crosstalk with other signaling pathways, thereby coordinating processes such as root apical meristem (RAM) maintenance (Yamada et al., 2020; Hsiao et al., 2025).

Peptide hormones also serve as upstream triggers of phosphorylation cascades that relay signals from the PM to the nucleus. Secreted peptides are perceived by PM-localized RLKs, which activate intracellular signaling relays, including MAPK cascades (Sun and Zhang, 2022; Ma and Nicolet, 2023). Activated receptor complexes amplify signals through a sequence of phosphorylation events, ultimately modulating nuclear transcription factors and cell-cycle regulators, including cyclins. These peptide-triggered signaling pathways collectively regulate cell proliferation, differentiation, and overall root growth (Lee et al., 2019; Yang et al., 2020b; Li et al., 2023).

Signaling pathways that directly regulate the physical properties of the cell wall are crucial for cell morphogenesis. Specific peptides alter cell wall plasticity by inducing the expression of genes involved in cell wall remodeling. Representative examples include *PGAZAT* (*pg abscission zone Arabidopsis thaliana*) and *PGLR* (*pg lateral root*) from the polygalacturonase family, *XTH23/XTR6* (*xyloglucan endotransglycosylase 6*) from the XTH family, and *EXP17* (*expansin 17*) from the expansin family. Alternatively, specific peptides can modulate the activity of cell-wall-relaxing enzymes by regulating extracellular pH (Kumpf et al., 2013; Zhu et al., 2019; Xu et al., 2020). Together, these mechanisms provide the necessary mechanical foundation for developmental processes that require cell wall relaxation, such as cell elongation and the emergence of lateral root primordia (LRP) (Lv et al., 2021; Shang et al., 2025).

These diverse intracellular signaling pathways do not operate in isolation but are intricately interwoven, forming a complex signaling network. Many of these pathways ultimately converge on nuclear regulatory mechanisms that control the expression, protein stability, or subcellular localization of key transcription factors such as PLETHORA (PLT) and root initiation transcription factor 1 (Yamada et al., 2020). This precise reprogramming of gene expression maintains RAM activity, shapes RSA, and fine-tunes plant environmental adaptation responses at the whole-root-system level.

Notably, the intricate signaling networks activated by peptide hormones often form a higher-dimensional regulatory hierarchy through crosstalk with classical plant hormones. For instance, certain peptide hormones regulate polar auxin transport and distribution, thereby influencing root gravitropism and LR formation (Liu et al., 2013; Aldowigh et al., 2025; Moore et al., 2025). Under conditions of nitrogen deficiency or excess, some peptide hormones interact with CK and other hormonal signals to coordinate RSA and optimize nutrient uptake (Taleski et al., 2023; Keerthana et al., 2025). Furthermore, peptide hormone signaling cooperates with brassinosteroid signaling to regulate cell wall synthesis and cell expansion (Xiao et al., 2025). This extensive signal integration tightly couples the rapid, localized signaling mediated by peptide hormones with the systemic, long-lasting effects of classical plant hormones, significantly expanding the regulatory repertoire through which plants coordinate development and adapt to fluctuations in nitrogen availability.

## Peptide hormones in primary root growth

The primary root, the main axis of the initial root system, develops from the embryonic radicle and plays a crucial role in anchoring the plant to the soil, absorbing water and nutrients, and responding to adverse conditions. Its continuous growth depends on the activity of the RAM, which consists of the quiescent center (QC) and surrounding stem cell populations that collectively constitute the stem cell niche (SCN) (Xie et al., 2023; Liu et al., 2024a, 2024b). QC cells maintain a low mitotic state and preserve the undifferentiated state of the surrounding stem cells. These multipotent stem cells undergo coordinated cell division and differentiation to generate tissues such as the root cap, epidermis, cortex, endodermis, and stele, thereby driving both longitudinal growth and radial expansion of the root (Chang et al., 2024; Liu et al., 2024a). The size and activity of the RAM are dynamically regulated by the balance between the rates of cell division and differentiation. This tightly controlled process is orchestrated by multiple core transcription factors and signaling pathways, including WUSCHEL-related homeobox 5 (WOX5), PLT, scarecrow (SCR), and short-root (SHR). In addition, peptide hormones provide local and long-distance signaling inputs that integrate these regulatory networks with systemic cues, thereby fine-tuning the balance between meristematic cell proliferation and differentiation and sustaining continuous root growth (Strotmann and Stahl, 2021; Yamoune et al., 2021).

### Peptides that regulate QC and RAM activity

**Figure 2:**
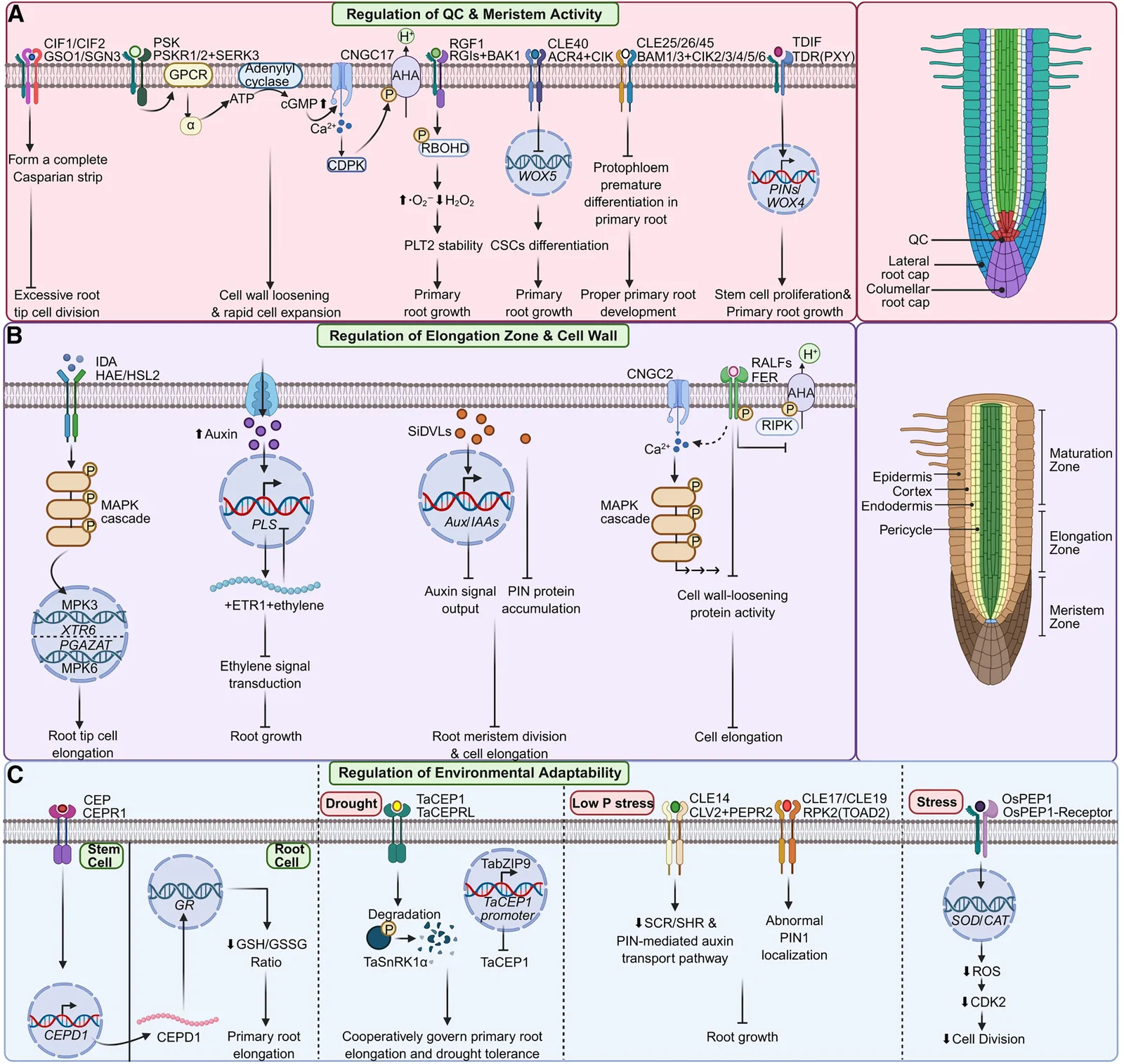
Peptide hormone regulatory networks controlling primary root growth. **(A)** Regulation of QC stability and RAM activity. Multiple peptide signals coordinately regulate stem cell fate and meristem maintenance. CLE40 binds to its receptor complex to repress *WOX5* expression, thereby balancing stem cell self-renewal and differentiation. The RGF1–RGI–BAK1 signaling cascade sustains the graded distribution of PLT transcription factors, maintaining the normal structure and size of the RAM. PSK activates downstream MAPK signaling and the PM H^+^-ATPase to promote cell expansion in the meristematic region. CIFs regulate Casparian strip formation to restrict excessive cell division, whereas TDIF modulates polar auxin transport and vascular stem cell activity to sustain root growth. **(B)** Modulation of root elongation and cell wall relaxation. Antagonistic peptide signals dynamically control cell wall remodeling and root elongation. The growth-promoting peptide IDA triggers HAE/HSL2 dimerization and subsequent MAPK activation, inducing the expression of cell wall hydrolases to loosen the cell wall and promote cell expansion. PLS mediates crosstalk between auxin and ethylene signaling, thereby relieving growth inhibition. In contrast, the RALF1–FER module suppresses PM proton pumping and cell wall acidification. It also increases cytosolic Ca^2+ levels^ and activates downstream MAPK signaling, thereby maintaining cell wall rigidity and restraining excessive cell elongation. **(C)** Peptide-mediated environmental adaptation of primary roots. Peptide hormones act as critical signals that enable roots to respond to nutrient fluctuations and abiotic stress. Long-distance CEP–CEPD systemic signaling adjusts root proliferation patterns in response to heterogeneous nitrogen distribution. Phosphorus-deficiency stress induces CLEs to alter auxin signaling and the SCR/SHR pathways, arresting meristem growth. PEP family peptides regulate ROS homeostasis and cell-cycle progression, helping roots cope with nutrient deficiency and immune stress. BAK1, BRI1-associated receptor kinase 1; HAE, HAESA; HSL2, HAESA-LIKE 2; PLS, POLARIS; PLT, PLETHORA; QC, quiescent center; RAM, root apical meristem; RGF1, root meristem growth factor 1; RGI, RGF1 insensitive; SCR, SCARECROW; SHR, SHORT-ROOT; TDIF, tracheary element differentiation inhibitory factor; WOX5, WUSCHEL-RELATED HOMEOBOX 5.

The WOX5 transcription factor serves as a central component of the dynamic molecular regulatory network operating within the QC and RAM. Specifically, *WOX5* is transcribed in the QC and directly suppresses the expression of *CYCD3;3* and *CYCD1;1*, thereby constraining mitotic activity in QC cells and preserving their quiescent state (Forzani et al., 2014). Moreover, WOX5 can move to neighboring columella stem cells, where it recruits the TOPLESS–HDA19 complex to induce histone deacetylation at the *CDF4* promoter, thereby stably suppressing stem cell differentiation (Zhang et al., 2025b). Reversible phosphorylation dynamically regulates WOX5 stability: an *Arabidopsis* shaggy-like kinase (AtSK) phosphorylates WOX5 and thereby stabilizes the protein, whereas dephosphorylation by a protein phosphatase 2A catalytic subunit promotes WOX5 degradation, thereby maintaining QC homeostasis (Zhang et al., 2025b). Additionally, CLE40 signaling suppresses *WOX5 expression through the receptor kinases ACR4* and CLV1, with CIKs functioning as co-receptors, thereby promoting QC cell division and stem cell differentiation (Berckmans et al., 2020) (Figure 2A).

Beyond the WOX5-centered regulatory pathway described above, peptide hormones, such as PSK, RGF1, and CLE peptides, also play crucial roles in regulating RAM activity, collectively forming a complex signaling network (Figure 2A). PSK binds to its receptors, PSKR1/2, and the co-receptor SERK3, thereby activating a MAPK cascade and H^+^-ATPases (AHAs) to promote cell wall relaxation and root cell expansion. A parallel pathway involving GPA1, cGMP, CNGC17, and CDPKs converges on AHA activation, leading to cell wall acidification and rapid cell expansion (Ladwig et al., 2015; Yu et al., 2020a; Kaufmann et al., 2021; Hu et al., 2023; Yang et al., 2023; Wu et al., 2025). Consistent with the function of PSK in *Arabidopsis*, overexpression of wheat *TaPSK5* significantly promotes root elongation and increases crop yield (Geng et al., 2020). In cedar, overexpression of *ClPSK1* or *ClPSK2* enhances primary root elongation by upregulating root-development-related genes, such as *PLT1*, *PLT2*, and *WOX5*, indicating that PSK has a conserved role in regulating root development across diverse plant species (Wu et al., 2019) (Figure 2A).

In addition to PSK, RGF1 plays a crucial role in regulating RAM activity. RGF1 is recognized through its sulfated RxGG motif by RGF receptors (RGI1–RGI5), thereby promoting the heterodimerization of RGIs with BAK1 (Kaufmann and Sauter, 2019). This signaling module contributes to RAM maintenance and root growth. In particular, RGF signaling promotes PLT accumulation and gradient formation. PLT transcription factors are master regulators of stem cell activity and meristem organization (Yamada et al., 2020). In *Arabidopsis*, the combined loss of RGF1–RGF3 disrupts the PLT gradient and reduces RAM size (Ou et al., 2016). Collectively, these findings indicate that RGF1–RGI–BAK1 signaling is essential for maintaining the PLT gradient and RAM integrity, although its downstream molecular mechanisms remain to be fully elucidated (Figure 2A).

In crop species, treatment with synthetic maize ZmRGF1 and tomato SlRGF1 peptides significantly increases RAM size and promotes primary root elongation (Fang et al., 2021b). The rice homolog *OsRGF1* is predominantly expressed in root tips. Loss of OsRGF1 function results in significantly shortened roots, whereas exogenous application of RGF peptide to the *Osrgf1* mutant restores meristematic activity and rescues the short-root phenotype (Fang et al., 2021b).

CLE peptides regulate RAM activity through diverse receptor-binding mechanisms. CLE40 binding to ACR4 promotes the interaction of ACR4 with CIK coreceptors and subsequent receptor phosphorylation, thereby balancing root stem cell maintenance and differentiation through the repression of *WOX5* expression. Furthermore, CLE25/26/45 bind to BAM1/3 receptors and CIK2/3/4/5/6 co-receptors to form signaling complexes. Acting downstream of the developmental regulators brevis radix and OCTOPUS, this pathway supports normal primary root development by preventing premature protophloem differentiation (Hu et al., 2022; Xiao et al., 2025). During early embryogenesis, the *CLE8* gene is expressed in both the endosperm and embryo, where it regulates the balance between cell division and differentiation. Mutant analysis reveals that loss of *CLE8* causes embryonic developmental arrest, reduced endosperm cell numbers, and premature maturation, highlighting its essential role in early embryonic development (Fiume and Fletcher, 2012). In rice, treatment with the OsCLE402/fon2-like cle protein 2 (FCP2) peptide suppresses the expression of *QHB*, a *WOX5* ortholog, thereby terminating RAM activity and impairing late metaxylem formation. Conversely, inducible overexpression of *QHB* alleviates the root growth inhibition caused by FCP2, indicating that the FCP2–QHB module plays a crucial role in maintaining the rice RAM and regulating late metaxylem development (Chu et al., 2013). Finally, treatment with TaCLE3p or TaCLE34p shortens primary roots in both wheat and *Arabidopsis*, suggesting that these peptides and their orthologs may have conserved roles in regulating primary root growth across monocots and dicots (Li et al., 2019) (Figure 2A).

CIF1 and CIF2, members of the CIF family, constrain cell division at the root tip by promoting the formation and maintenance of the Casparian strip in the root endodermis. CIFs are sulfated peptides secreted by the root vasculature that specifically bind to the LRR-RLK receptor GASSHO1/SCHENGEN3 (SGN3) on the surfaces of endodermal cells. This interaction triggers SGN3-dependent signaling, promoting the formation and maintenance of the endodermal Casparian strip. The Casparian strip acts as a physical barrier, limiting the diffusion of cell division signals, such as auxin, and thereby preventing excessive cell division at the root tip (Jeon et al., 2021).

Another peptide that regulates root tip activity is tracheary element differentiation inhibitory factor (TDIF, also known as CLE41 or CLE44), which promotes primary root growth. This effect does not depend on changes in auxin biosynthesis or catabolism but instead results from a reduction in the auxin maximum at the root tip (Yang et al., 2020a). Mechanistic studies have revealed that TDIF specifically upregulates the transcription of *PIN genes, whereas several pin* single mutants exhibit insensitivity to TDIF treatment. These findings suggest that TDIF-induced primary root growth depends on PIN-mediated polar auxin transport (Yang et al., 2020a). Furthermore, the TDIF–TDR (also known as PXY) peptide–receptor signaling pathway activates the transcription factor WOX4, thereby maintaining cambial stem cell identity and promoting stem cell proliferation. This enables the continuous production of new vascular cells while preventing depletion of the stem cell pool through excessive differentiation (Wang et al., 2025b; Zhang et al., 2025d).

In addition to TDIF, the small peptide miPEP165a, encoded by pri-miR165a in *Arabidopsis*, also enhances primary root growth. Following exogenous application, miPEP165a initially enters cells through passive diffusion in the root cap and meristematic zones, whereas in the differentiation and mature zones, it is internalized through clathrin-mediated endocytosis and membrane microdomain-associated endocytosis. Once inside the cell, miPEP165a enhances the expression of its own primary transcript, pri-miR165a, through a positive autoregulatory feedback loop, thereby increasing the accumulation of mature miR165a. Subsequently, miR165a suppresses the expression of genes encoding HD-ZIP III transcription factors, thereby alleviating their inhibitory effects on cell division in the RAM. The resulting increase in cell proliferation and cell number ultimately promotes root growth (Ormancey et al., 2020) (Figure 2A).

### Peptides that regulate root elongation and cell wall relaxation

In the root elongation zone, the peptide hormone IDA promotes heterodimerization between the receptors HAESA (HAE) and HAE-LIKE2 (HSL2), thereby activating the downstream MAPK cascade. Activated MPK3 and MPK6 induce the expression of the cell-wall-remodeling genes *XTR6* and *PGAZAT*, thereby facilitating cell elongation and root growth (Kumpf et al., 2013; Zhu et al., 2019; Xu et al., 2020) (Figure 2B).

In *Arabidopsis*, POLARIS (PLS) is a 36-amino-acid peptide expressed in the root-tip region where auxin accumulation is maximal. The loss-of-function mutant *pls* exhibits a SHR phenotype, whereas exogenous application of synthetic PLS peptide rescues this phenotype (Chilley et al., 2006). Further studies showed that auxin accumulation in the root tip directly induces *PLS* transcription. Moreover, PLS can interact with the ethylene receptor ETR1, thereby relieving ethylene-mediated repression of *PLS* transcription and establishing a positive-feedback loop (Chilley et al., 2006). This interaction was subsequently shown to alleviate ethylene-mediated inhibition of root growth (Mudge et al., 2025). However, the molecular mechanisms by which PLS promotes root cell elongation remain unclear (Figure 2B).

SiDVL peptides, identified in *Setaria italica*, also inhibit root elongation. Overexpression of *SiDVL* genes in *Arabidopsis* resulted in significantly shorter roots. Further analysis showed that SiDVL reduces auxin signaling output by upregulating *Aux*/*IAA* expression and disrupts polar auxin transport by reducing PIN accumulation. Together, these effects suppress cell division in the RAM and cell elongation in the elongation zone (Wang et al., 2024) (Figure 2B).

Unlike growth-promoting peptides, RALF1 strongly inhibits cell-wall expansion and consequently restricts primary root elongation. RALF1 binding to FERONIA (FER) activates the FER–RIPK signaling complex, which phosphorylates AHA2, reducing proton extrusion and apoplastic acidification (Haruta et al., 2014; Du et al., 2016). The resulting alkalinization limits the activity of cell-wall-remodeling proteins, including pectin methylesterases, and reinforces cell-wall rigidity (Rossling et al., 2024). In parallel, the RALF1–FER module elevates cytosolic Ca^2+^ levels and activates the MAPK cascade, further amplifying the growth-inhibitory signal. Together, reduced cell-wall acidification and sustained wall rigidity constrain cell elongation. Thus, the RALF–FER module plays a central role in cell-wall-integrity sensing and growth regulation in *Arabidopsis* root tips and epidermal cells (Zhang et al., 2023a) (Figure 2B).

### Peptides that regulate primary root environmental adaptability

Plants can adjust their RSA in response to spatial variation in soil nitrogen availability. In this process, CEPs and their downstream signaling molecules, CEP downstream regulators (CEPDs), play pivotal roles. Specifically, the binding of CEP1 to its receptor CEPR1 activates a downstream signaling cascade that induces *CEPD1* expression in shoots. CEPD1 is a mobile polypeptide belonging to the plant-specific glutathione-like protein family. Following its transport to the roots, CEPD1 modulates nitrogen uptake and root growth, thereby enabling preferential root proliferation in nitrogen-rich zones (Taleski et al., 2023) (Figure 2C).

In wheat, TaCEP1 also contributes to the regulation of RSA by modulating primary root growth through a mechanism associated with drought adaptation. Recognition of TaCEP1 by the receptor kinase TaCEPRL promotes the phosphorylation and degradation of TaSnRK1α, thereby attenuating its positive effects on primary root elongation and drought tolerance. Meanwhile, the transcription factor TabZIP9 directly binds to the *TaCEP1* promoter, repressing *TaCEP1* transcription and thereby coordinating primary root growth with drought adaptation (Wen Yang et al., 2025) (Figure 2C).

Phosphate deficiency induces *CLE14* expression in the proximal root meristem, where the resulting CLE14 peptide promotes RAM differentiation through the receptors CLV2 and PEPR2, thereby arresting primary root growth. Further analyses indicate that the CLE14–CLV2/PEPR2 regulatory module mediates plant adaptation to phosphate deficiency by modulating auxin signaling and the SCR/SHR pathways (Gutierrez-Alanis et al., 2017). Additionally, CLE17/CLE19-mediated regulation of cell proliferation in the RAM via the RPK2 receptor, also known as TOAD2, involves auxin signaling, as *rpk1 rpk2 double mutants exhibit* abnormal PIN1 localization and reduced activity of *DR5*, a synthetic auxin-responsive promoter (Jeon et al., 2021) (Figure 2C).

Another class of important environmental sensors, PEPs, regulates primary root growth by integrating environmental signals. Specifically, OsPEP1 binds to an unidentified receptor and activates downstream signaling pathways, promoting the expression of *superoxide dismutase* and *catalase* and thereby reducing ROS levels in root tips. Reduced ROS levels decrease cyclin-dependent kinase 2 (CDK2) activity, preventing cells from entering S phase and thereby suppressing cell division in the root meristem (Xiang et al., 2021; Kirova et al., 2022). Furthermore, other immune-related peptide hormones in *Arabidopsis* also regulate primary root growth under stress. For example, SCOOP12 activates immune responses and modulates root growth by directly binding to MIK2 and promoting the formation of a receptor complex with BAK1 (Hou et al., 2021; Rhodes et al., 2021).

In summary, peptide hormones precisely coordinate primary root development by integrating stimulatory and inhibitory regulatory programs. The inhibitory system operates through three key strategies: disrupting RAM activity to limit stem cell proliferation, suppressing cell wall loosening and expansion to directly restrict cell growth, and creating physical and molecular barriers that limit the movement of auxin and other growth-promoting signals. Together, these mechanisms ensure optimal resource allocation and developmental stability during primary root elongation. In contrast, the positive regulatory system promotes primary root elongation at multiple levels by facilitating cell wall remodeling and cell expansion, maintaining RAM activity, and regulating auxin transport and distribution (Figure 2C). At the systems level, although antagonism between positive and negative regulatory signals underpins many of these pathways, the two systems also interact dynamically to coordinate core processes, including auxin distribution, ROS homeostasis, cell wall properties, and the balance between cell division and differentiation. These interactions enable adaptive control of primary root elongation, meristem activity, and developmental timing. This bidirectional regulatory network is exemplified by RGF1–ROS signaling-mediated stabilization of PLT2, CLE–MAPK cascade-mediated repression of WOX5, the antagonistic effects of auxin and abscisic acid (ABA) on *PIN* gene expression, and feedback loops linking peptide hormone signaling to ROS homeostasis. Collectively, these mechanisms enable plants to flexibly adjust RSA in response to internal developmental signals, such as auxin gradients and meristematic cues, as well as external environmental cues, including nutrient availability and water status. These interconnected regulatory nodes provide precise molecular targets for molecular breeding strategies aimed at developing crops with optimized RSA, allowing breeders to fine-tune root growth, resource allocation, and stress resilience under fluctuating field conditions.

## Peptide hormones in LR formation

**Figure 3:**
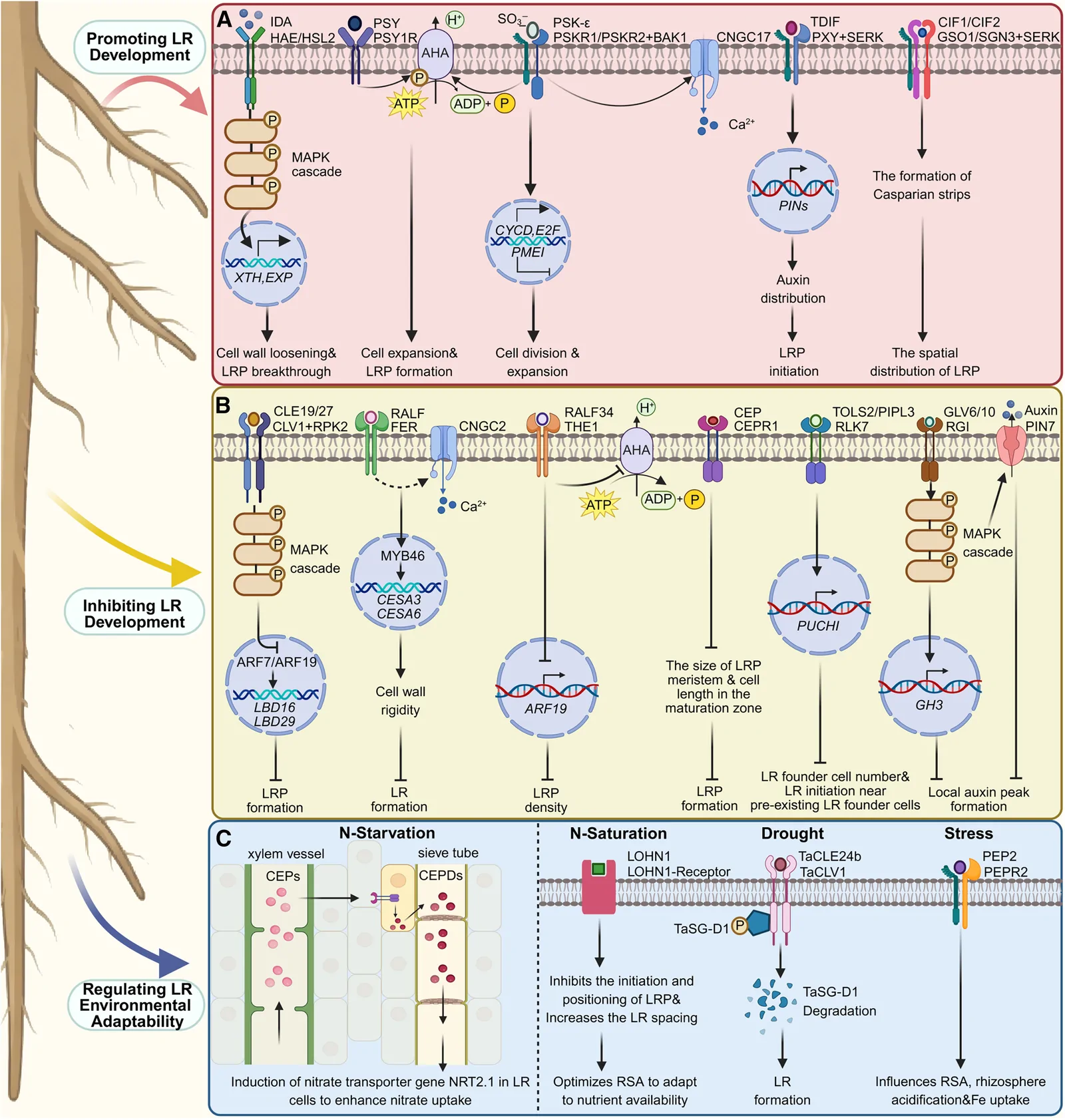
Peptide hormones precisely regulate LR development and environmental adaptation. **(A)** Peptide hormones that promote LR development. Peptide hormones drive LR initiation and emergence through distinct mechanisms. The IDA–HAE/HSL2 module activates a MAPK cascade that upregulates cell-wall-loosening genes encoding XTHs and EXPs, thereby facilitating lateral root primordium emergence through the overlying tissues. PSY1 and sulfated PSK-ε enhance cell expansion through AHA2-mediated proton pumping and cell-cycle activation, respectively. TDIF modulates auxin distribution by upregulating PIN transporters, thereby priming LR initiation sites, whereas CIFs maintain Casparian strip integrity and help ensure the proper positioning of emerging primordia. **(B)** Peptide hormones that inhibit LR development. Antagonistic signals prevent excessive LR branching and thereby optimize resource allocation. CLE peptides (CLE19/27) suppress LR initiation via CLV1/RPK2–MAPK cascades that inhibit the auxin-responsive transcription factors ARF7/19. RALF peptides (RALF1/34) increase cell-wall rigidity through FER/CNGC2- or THE1-mediated pathways, whereas GLVs deplete local auxin maxima by promoting PIN7-mediated efflux and GH3-mediated auxin conjugation. CEP and TOLS2 signals further reduce meristem size and founder-cell number to maintain appropriate LR spacing. **(C)** Peptide-mediated environmental adaptation of LRs. Peptide signals remodel LR development in response to changes in the soil environment. Under nitrogen starvation, root-derived CEPs trigger systemic nitrogen-demand signaling that induces nitrate-transporter expression in LRs. Nitrogen-replete conditions activate LOHN1 to adjust LR spacing. In wheat, the drought-responsive peptide TaCLE24b promotes LR formation, whereas stress-induced PEP2 modulates rhizosphere acidification and iron uptake to enhance environmental adaptation. Collectively, these modules enable plants to flexibly adjust LR density and distribution to maximize resource acquisition under fluctuating environmental conditions. AHA2, *Arabidopsis* H^+^-ATPase 2; ARF7/19, auxin response factors 7 and 19; CEP, C-terminally encoded peptide; CIF, Casparian strip integrity factor; CLE, CLAVATA3/EMBRYO SURROUNDING REGION-related; CLV1, CLAVATA1; CNGC2, cyclic nucleotide-gated channel 2; EXP, expansin; FER, FERONIA; GH3, Gretchen Hagen 3; GLV, GOLVEN; HAE, HAESA; HSL2, HAESA-LIKE 2; LOHN1, LATERAL ROOT OVERPRODUCTION UNDER HIGH-NITROGEN CONDITIONS 1; LR, lateral root; MAPK, mitogen-activated protein kinase; PEP2, plant elicitor peptide 2; PIN, pin-formed; PSK, phytosulfokine; PSY1, plant peptide containing sulfated tyrosine 1; RALF, rapid alkalinization factor; RPK2, receptor-like protein kinase 2; TDIF, tracheary element differentiation inhibitory factor; THE1, THESEUS1; TOLS2, TARGET OF LBD SIXTEEN 2; XTH, xyloglucan endotransglucosylase/hydrolase.

LRs are branch roots that arise from the primary root. By expanding the root system surface area and soil exploration capacity, they enhance water and nutrient uptake, anchorage, and stress tolerance, thereby representing a core manifestation of RSA plasticity (Zhang and Shang, 2024). In *Arabidopsis*, LR formation begins with the division of xylem-pole pericycle cells, followed by asymmetric divisions that generate an LRP. The primordium subsequently establishes a functional root meristem and emerges through the overlying endodermal, cortical, and epidermal cell layers (Beckers et al., 2025). Auxin acts as a central regulator by activating the transcription factors ARF7/19, which induce downstream genes involved in cell-cycle progression and cell-wall remodeling, thereby promoting LR emergence (Yu et al., 2022b, 2024; Zhang et al., 2022; Shang et al., 2025). Most peptide hormones regulate LR development through direct or indirect interactions with auxin signaling. Based on their functional roles, these signaling modules can be systematically categorized into three groups: those that promote LR development, those that suppress LR development, and those that mediate adaptive responses to environmental conditions (Figure 3).

### Peptides that promote LR development

Certain peptide hormones positively regulate LR development. For example, binding of IDA to its receptors HAE and HSL2 activates the MAPK signaling cascade, which upregulates genes involved in cell-wall remodeling, including members of the *XTH* and *EXP* families. This promotes cell wall relaxation, a crucial step for LRP to penetrate the epidermis. Mutants lacking *IDA*, *HAE*, or *HSL2* exhibit arrested LRP development at stages III and IV and significantly reduced LR density (Kumpf et al., 2013; Zhu et al., 2019; Xu et al., 2020) (Figure 3A).

PSY1, a tyrosine-sulfated peptide, signals through its receptor PSY1R, which phosphorylates AHA2 and thereby promotes H^+^ extrusion and apoplastic acidification. The resulting increase in cell expansion facilitates LRP formation (Jeon et al., 2021). The *psy1r* mutant exhibits impaired primary root elongation and reduced LR density, whereas the exogenous application of PSY1 partially restores these phenotypes (Hsiao and Yamada, 2020; Tost et al., 2021). In contrast, the maize *PSY-family peptide* homolog *ZmPSY3* is specifically expressed in roots under abiotic stress and regulates stress-induced carotenoid biosynthesis (Li et al., 2008). ABA, which is derived from carotenoids, can subsequently inhibit LR initiation by regulating the polar localization of ZmPIN1, thereby altering auxin distribution (Lu et al., 2019). Together, these findings suggest that the effects of PSY1-mediated regulation on LR development may differ among plant species (Figure 3A).

PSK-ε exerts a potent stimulatory effect on LR development. In *Medicago truncatula*, exogenous application of 1 μM sulfated PSK-ε peptide for 20 days doubled the number of LRs. Moreover, overexpression of *MtPSKε* in *M. truncatula* increased LR number by approximately 78%, and this effect was conserved upon heterologous expression in *Arabidopsis* (Di et al., 2022). Following sulfation by TPST, the sulfated PSK-ε is perceived by PSKR1/2 receptors in conjunction with the co-receptor BAK1. Activation of this receptor complex promotes LR development through two complementary mechanisms: (1) activation of AHA1/2 proton pumps and CNGC17 ion channels and (2) transcriptional induction of cell-cycle genes (e.g., *CYCD* and *E2F*) coupled with repression of *PMEI*, a negative regulator of cell expansion (Ladwig et al., 2015) (Figure 3A).

Like GLVs, CIFs can also regulate the spatial positioning of LRPs; however, CIFs differ from GLVs in that they primarily monitor and maintain the integrity of the endodermal Casparian strip. In *Arabidopsis*, CIF1 and CIF2 are peptide hormones containing sulfated tyrosine, expressed in the stele and secreted into the apoplast. Upon binding to the receptor kinase GASSHO1/SGN3 at the endodermal plasma membrane, CIFs promote the formation of a complex with SERK co-receptors and the subsequent phosphorylation of downstream substrates. This signaling cascade promotes the assembly of Casparian strip membrane domain proteins into continuous microdomains at the endodermal PM (Okuda et al., 2020). In the *cif1 cif2* double mutant, defects in the Casparian strip compromise the root ion-diffusion barrier. Consequently, LRPs lose spatial guidance during endodermal penetration, resulting in disorganized emergence and significantly reduced LR density (Doll, 2024; Zhang et al., 2024b). In rice, OsCIF1 and OsCIF2 similarly contribute to Casparian strip formation in the endodermis, thereby influencing the spatial positioning of LRPs. These findings indicate that the CIF-mediated establishment of endodermal barriers and of LR patterning are conserved across species (Ren et al., 2024) (Figure 3A).

TDIF plays a pivotal role in auxin-dependent LRP initiation. It enhances local auxin accumulation at LRP initiation sites along the longitudinal axis of the primary root without affecting auxin biosynthesis or metabolism (Yang et al., 2020a). Mechanistic studies reveal that TDIF promotes polar auxin transport by upregulating *PIN* gene transcription (Yang et al., 2020a), underscoring its critical role in shaping the spatial distribution of auxin and thereby precisely guiding LRP initiation (Figure 3A).

### Peptides that inhibit LR development

LR development is continuously adjusted to accommodate intrinsic functional demands and changing environmental conditions. Peptide hormones that restrict LR formation and thereby prevent excessive resource expenditure play a crucial role in this process (Figure 3B). Within the CLE family, *CLE19* and *CLE27* are specifically expressed in LRP-associated cells, particularly pericycle cells and adjacent cortical cells, as revealed by promoter-driven GUS reporter assays (Czyzewicz et al., 2015). Exogenous application of 10 μM synthetic CLE19 peptide reduces LR formation by 40%–50% and causes LRP development to arrest at stages III and IV. Further studies indicate that CLE19 activates a MAPK cascade by binding to the receptors CLV1 and RPK2. The activated MAPK subsequently translocates to the nucleus, where it inhibits the activities of ARF7 and ARF19 and downregulates genes associated with LR initiation , including *LBD16* and *LBD29* (Fiers et al., 2005; Lee et al., 2019). CLE27 appears to act through a similar pathway and, at the relatively high concentration of 20 μM, completely blocks LRP formation (Czyzewicz et al., 2015) (Figure 3B).

RALFs represent another class of peptide hormones that strongly inhibit LR formation. Disruption of their receptor FER results in increased LR numbers and more rapid LRP emergence. Mechanistically, RALFs bind to FER, triggering Ca^2+^ influx through the putative calcium channel CNGC2. This signaling cascade upregulates cellulose synthase genes, including *CESA3* and *CESA6*, via the transcription factor MYB DOMAIN PROTEIN 46 (MYB46), ultimately increasing cell-wall rigidity. Concurrently, FER modulates auxin distribution by regulating the polar localization and endocytic cycling of PIN2. Together, these processes suppress LR development (Wang et al., 2017; Dong et al., 2019; Kamon et al., 2021; Chen et al., 2025). RALF34 signals through the receptor THE1; *the1* mutant seedlings exhibit an approximately 30% increase in LR density. This mechanism involves inhibition of AHA2 by the RALF34–THE1 complex, increasing apoplastic pH and reducing cell-wall relaxation and *ARF19* transcription (Bacete et al., 2022; Li et al., 2025) (Figure 3B).

CEPs are induced under conditions of abundant sucrose availability and subsequently inhibit LR growth through the receptor CEPR1 by reducing size of the LR meristematic zone and cell length in the maturation zone. Exogenous application of CEP5 significantly suppresses LR growth in wild-type plants but has no effect on *cepr1* seedlings (Chapman et al., 2019). Interestingly, CEPs also function as systemic signals of nitrogen deficiency, promoting nitrogen uptake by upregulating nitrate transporters and facilitating nodule formation in legumes (Taleski et al., 2018; Teyssendier de la Serve et al., 2026). These findings suggest that CEPs may play a crucial role in coordinating LR development with plant carbon and nitrogen status (Figure 3B).

The GLV peptides, primarily GLV6 and GLV10, inhibit LR formation by preventing the localized auxin accumulation required for LRP initiation. Overexpression of *GLV6* or *GLV10*, as well as exogenous application of their corresponding synthetic peptides, markedly suppresses LR formation, thereby reducing LR density and increasing the spacing between LRs (Fernandez et al., 2020). Mechanistically, GLV signaling through RGI receptors activates a MAPK cascade that simultaneously upregulates the auxin efflux carrier PIN7 and the auxin-conjugating enzyme GH3. This dual action depletes auxin at LR initiation sites by enhancing auxin efflux and metabolism. Notably, GLV10 also specifically regulates the spatial distribution of LRs, shifting the position of the first LR toward the root tip and significantly shortening the LR formation zone. This regulatory effect is strictly dependent on functional RGFR family receptors (Jourquin et al., 2023; Doll, 2024). In peach trees, the GLV-like peptide pCTG134 synergistically activates auxin and ethylene biosynthesis and signaling by upregulating the ethylene receptor gene *ETR1*, the transcription factor gene *EIN3*, genes involved in auxin biosynthesis, and the auxin efflux carrier gene *PIN2*. Although ethylene typically inhibits LR formation, pCTG134 substantially promotes LR development (Busatto et al., 2017). Nevertheless, the molecular mechanism by which pCTG134 regulates LR formation and produces an effect distinct from that of its homologous GLV peptides remains unknown (Figure 3B).

The TOLS2 peptide, which is expressed in LR founder cells, acts through its receptor RLK7 and the downstream transcription factor PUCHI to inhibit the specification of adjacent pericycle cells, thereby eliminating supernumerary founder cells and ensuring proper LR spacing (Toyokura et al., 2019). In *Arabidopsis*, miPEP165a, a peptide hormone translated from the microRNA precursor transcript *pri-miR165a*, suppresses LR formation while promoting primary root growth; similarly, miPEP171b suppresses LR formation in *M. truncatula* (Ormancey et al., 2020). Mechanistic studies have revealed that these miPEPs not only enhance the transcription of their corresponding primary microRNA transcripts, thereby increasing the accumulation of their mature microRNAs, but also suppress the expression of genes associated with LR development, collectively restricting LR formation (Ormancey et al., 2020) (Figure 3B).

### Peptide hormone regulation of LR adaptation to environmental cues

Beyond developmental signals, LR formation and elongation are strongly modulated by environmental cues. In this process, peptide hormones play a crucial role in interpreting environmental signals and fine-tuning LR adaptation. Under nitrogen starvation, root-derived CEPs translocate to the shoots, where they are recognized by CEPR1/2, triggering the synthesis of the downstream signaling peptides CEPD1/2. CEPD1/2 subsequently move back to the roots and activate the nitrate transporter NRT2.1, thereby enhancing nitrate uptake (Ahmad et al., 2023). Thus, the upstream CEPs and downstream CEPDs coordinately mediate systemic root-to-shoot-to-root signaling in response to nitrogen deprivation (Figure 3C). Additionally, CEPs play crucial roles in regulating LR responses to nitrogen availability in crops. In alfalfa, for example, MtCEP1 inhibits LR formation through the receptor compact root architecture 2 (MtCRA2) (Taleski et al., 2018); in rice, both exogenous application of synthetic OsCEP6.1 peptide and the transgenic overexpression of *OsCEP6.1* inhibit LR development by modulating nitrogen-responsive signaling (Sui et al., 2016) (Figure 3C).

In contrast to CEPs, the peptide hormone LOHN1 acts as a systemic nitrogen-saturation signal that regulates LR spacing. LOHN1 is expressed and secreted in the stele. Through a receptor-mediated mechanism that has yet to be fully elucidated, it restricts both the initiation and spatial positioning of LRPs, thereby increasing LR spacing. *lohn1* mutants exhibit significantly increased LR density and reduced spacing under nitrogen-saturated conditions, whereas exogenous application of a synthetic LOHN1 peptide restores increased LR spacing. As a downstream effector of systemic nitrogen-saturation signaling, LOHN1 prevents excessive root branching when nitrogen is abundant, thereby optimizing RSA in response to nutrient availability (Ito et al., 2025) (Figure 3C).

A further example of species-specific peptide regulation is provided by TaCLE24b in wheat. Upon binding to its receptor TaCLV1, TaCLE24b enhances the interaction between TaCLV1 and TaSG-D1, leading to TaSG-D1 phosphorylation and degradation. This process subsequently promotes LRP formation. Sustained activation of this pathway also significantly enhances wheat survival under drought conditions by coordinating LR development, stomatal closure, and the maintenance of chlorophyll content, demonstrating how peptide hormones coordinately regulate growth and stress responses (Yang et al., 2025; Li et al., 2026) (Figure 3C).

In addition to the aforementioned LR-regulatory peptides with well-defined molecular mechanisms, several other peptide hormones have been provisionally implicated in LR development. For instance, PEP2 influences RSA and LR adaptation to environmental conditions via its receptor PEPR2. The PEP2–PEPR2 module regulates rhizosphere acidification and iron uptake, thereby affecting iron accumulation and root development (Shikha et al., 2026). Collectively, these finely tuned regulatory networks ensure optimal LR distribution in accordance with intrinsic developmental programs and environmental conditions (Figure 3C).

In summary, peptide hormones precisely coordinate LR development through the integration of positive and negative regulatory systems. The negative regulatory system primarily operates through three major mechanisms: (1) directly interfering with the auxin signaling hub to block LR initiation by suppressing the activity of key transcription factors or reducing localized auxin accumulation; (2) altering cell-wall mechanical properties by enhancing cellulose synthesis or inhibiting the activity of cell-wall-loosening enzymes, thereby impeding LRP penetration; and (3) establishing physical and chemical signaling barriers by maintaining endodermal integrity or coupling nutrient signals with mechanisms that restrict the spatial distribution of LRs. Collectively, these mechanisms optimize resource allocation and ensure developmental precision in RSA. Positive regulation, by contrast, primarily promotes LR formation through two key mechanisms: (1) directly accelerating cell-wall remodeling to create the mechanical conditions required for LRP emergence and (2) activating cell-enlargement programs that provide the intrinsic force required for cell expansion. From a systems perspective, these positive and negative regulatory mechanisms do not simply antagonize each other. Instead, they interact dynamically to finely coordinate core processes, including auxin distribution, cell-wall properties, and cell division and differentiation. Together, these interactions enable the adaptive regulation of LR density, spatial distribution, and developmental timing. This bidirectional regulatory framework allows plants to flexibly adjust their RSA in response to internal developmental cues and external environmental signals and provides important genetic targets for improving crop RSA.

## Peptide hormones in root hair development

**Figure 4:**
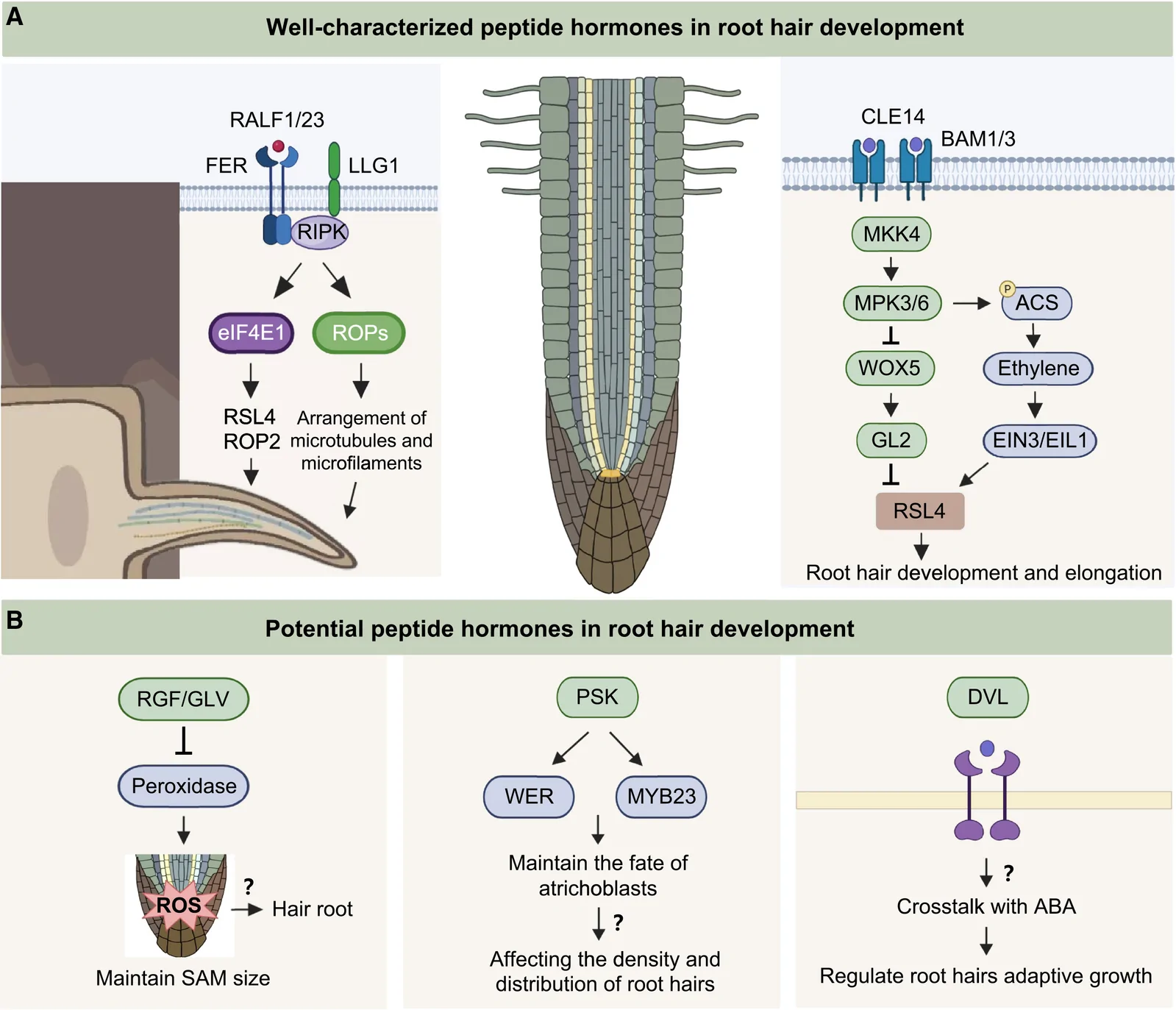
Regulation of root hair development by peptide hormones. **(A)** Well-characterized core peptide hormone pathways controlling root hair development. Left, the RALF1/23 pathway: RALF1/23 peptides are perceived by the PM-localized FER–LLG1–RIPK receptor complex, activating downstream ROP GTPases, which regulate cytoskeletal organization, and eIF4E1, which enhances the translation of growth regulators such as RSL4, thereby promoting polarized root hair growth. Right, the CLE14 pathway: CLE14 is recognized by BAM1/3 receptors, triggering an MKK4–MPK3/6 cascade that represses *WOX5*, reduces GL2 activity, and relieves the repression of RSL4, thereby promoting root hair growth. This pathway also enhances ethylene signaling through ACS phosphorylation, further reinforcing RSL4 activity. **(B)** Potential peptide hormone regulators of root hair development. RGF/GLV peptides may maintain root apical meristem activity through ROS-mediated signaling, thereby indirectly influencing root hair development. PSK may modulate epidermal cell fate by regulating WER and MYB23, potentially altering root hair density. DVLs may interact with ABA signaling to mediate adaptive root hair growth under stress. Collectively, these peptides integrate developmental and environmental cues to fine-tune root hair patterning, elongation, and adaptation to stress. ABA, abscisic acid; ACS, 1-aminocyclopropane-1-carboxylate synthase; BAM1/3, BARELY ANY MERISTEM 1 and 3; DVL, DEVIL; eIF4E1, eukaryotic translation initiation factor 4E1; FER, FERONIA; GL2, GLABRA2; GLV, GOLVEN; LLG1, LORELEI-LIKE GPI-ANCHORED PROTEIN 1; MKK4, mitogen-activated protein kinase kinase 4; MPK3/6, mitogen-activated protein kinases 3 and 6; PSK, phytosulfokine; RALF, rapid alkalinization factor; RGF, root meristem growth factor; RIPK, RPM1-INDUCED PROTEIN KINASE; ROP, Rho of Plants GTPase; ROS, reactive oxygen species; RSL4, ROOT HAIR DEFECTIVE 6-LIKE 4; WER, WEREWOLF; WOX5, WUSCHEL-RELATED HOMEOBOX 5.

Root hairs are tubular extensions of specialized root epidermal cells that increase root–soil contact, thereby facilitating water and nutrient absorption, anchorage, and microbial interactions (Sui et al., 2016, 2025; Zhang et al., 2023b). During root hair development, epidermal cells differentiate into trichoblasts (hair-forming cells) and atrichoblasts (non-hair cells), a process controlled by transcription factors including WEREWOLF (WER), GLABRA2, and transparent testa glabra (Dolan and Costa, 2001; Grierson et al., 2014). Trichoblasts subsequently undergo tip growth, which depends on an apical Ca^2+^ gradient regulated by CPK1 and CNGC5/6/9, ROP GTPase-mediated cytoskeletal reorganization, and expansin-mediated cell-wall loosening (Molendijk et al., 2001; Lin et al., 2011; Zhu et al., 2025a). Recent studies have revealed that several peptide hormones are integral components of this regulatory network (Figure 4).

### Core peptide hormones regulating root hair development

Based on their regulatory roles and mechanisms of action, peptide hormones that regulate root hair development can be divided into the core group, which includes RALFs and CLEs, and the non-core group (Figure 4).

RALFs directly regulate polarized root hair growth. RALF1/23 are recognized by the receptor FER at the PM, which recruits the co-receptor LORELEI-like GPI-anchored protein 1 and activates RIPK, thereby initiating downstream signaling (Haruta et al., 2014; Stegmann et al., 2017; Xiao et al., 2019). This pathway promotes root hair tip growth by activating ROP GTPases (Huang et al., 2013) and phosphorylating eIF4E1, thereby enhancing the translation of key growth regulators, including RSL4 and ROP2 (Zhu et al., 2020b). RSL4, in turn, represses *RALF1* expression, forming a negative-feedback loop that prevents excessive root hair elongation. Additionally, FER functions upstream of RAC/ROP signaling to regulate ROS-mediated root hair development (Huang et al., 2013; Duan et al., 2020). Notably, the fungal pathogen *Fusarium oxysporum* secretes a RALF-like peptide (F-RALF) that hijacks this signaling module to inhibit root hair elongation (Masachis et al., 2016; Tan et al., 2024) (Figure 4A).

In contrast to RALFs, CLEs integrate long-distance signaling with developmental regulation. CLE14, produced in epidermal cells, moves to the stele, where it is perceived by BAM1/3 in pericycle cells. This activates a MAPK cascade that suppresses WOX5, thereby reducing *GLABRA2* expression and relieving the repression of RSL4 to promote root hair growth. Additionally, CLE14–MAPK signaling increases ethylene production through the phosphorylation of 1-aminocyclopropane-1-carboxylic acid (ACC) synthase and suppresses GTL1 through CLV2/CRN signaling, with both processes further enhancing RSL4 activity (Shibata et al., 2018; Kubenova et al., 2022; Martin et al., 2022; Zhou et al., 2025). Collectively, CLEs function as central regulators that coordinate receptor activation, MAPK cascades, ethylene signaling, and transcriptional reprogramming during root hair development (Figure 4A).

### Other candidate peptide hormones involved in root hair development

Apart from RALFs and CLEs, relatively few studies have directly linked peptide hormones to the regulation of root hair initiation, elongation, or density. However, given the extensive roles of peptide hormones in RAM maintenance, cell differentiation, and auxin signal transduction, other peptide families, including RGFs/GLVs, may indirectly influence root hair development. RGF1 increases ROS levels in the meristematic region by inhibiting peroxidase activity, thereby maintaining cell division, delaying differentiation, and ultimately enlarging the meristem (Matsuzaki et al., 2010; Meng et al., 2012; Whitford et al., 2012; Yamada et al., 2020). Studies have shown that impaired RAM function typically leads to stunted root growth and/or aberrant cell differentiation, which may consequently disrupt root hair development (Yamada et al., 2020). Therefore, RGFs/GLVs represent potential indirect regulators of root hair development, and the possible role of ROS in RGF/GLV-mediated root hair cell fate determination warrants further investigation (Figure 4B).

PSK also determines the fate of atrichoblasts (Kaufmann et al., 2021; Wu et al., 2025). Its precursor undergoes TPST-mediated tyrosine sulfation to generate the biologically active peptide (Matsubayashi et al., 2002; Komori et al., 2009; Okuda et al., 2020). PSK promotes the expression of *AtWER* and its homolog *AtMYB23*, which maintain atrichoblast identity, thereby indirectly affecting the density and distribution of root hairs. Notably, PSK receptor signaling can remain active even in the absence of TPST-mediated sulfation (Kaufmann et al., 2021). These findings indicate that the PSK–receptor signaling module plays an important role in atrichoblast fate specification. Consequently, under specific conditions, such as environmental stress, diminished PSK activity or downregulation of its cognate receptor proteins may perturb epidermal cell fate specification, thereby increasing the proportion or density of root hair-forming cells (Figure 4B).

The involvement of DVL peptides in root stress responses also suggests a potential role in root hair development. Abiotic stress induces *DVL* expression in root tip stem cells, and the resulting peptides bind their receptors to fine-tune ABA signaling, thereby preserving root tip cell viability and proliferative capacity (Yang et al., 2024a). Notably, plants commonly adapt to drought by altering root hair morphology and density to improve water and nutrient acquisition (Zhang et al., 2023b). Therefore, DVLs may contribute to the environmentally responsive regulation of root hair development through crosstalk with the ABA signaling pathway (Figure 4B).

Moreover, many peptide hormones have been shown to modulate auxin synthesis, transport, and signaling, thereby affecting root development (Chapman et al., 2020; Li et al., 2022). Given that auxin is a central promoter of root hair development—primarily by controlling root hair cell specification, initiation, and polarized elongation (Masucci and Schiefelbein, 1996; Cho et al., 2007; Zhang et al., 2023b)—any peptide hormone that alters auxin homeostasis or signal transduction may indirectly regulate root hair formation. For example, GLVs affect auxin distribution by altering the abundance and stability of PIN auxin transporters at the PM, further supporting their potential involvement in root hair development (Whitford et al., 2012).

Although evidence for the direct regulation of root hair development by peptide hormones remains limited, their well-established roles in other aspects of root development, together with their extensive interactions with classical phytohormone pathways, suggest that their contribution to root hair development is likely broader than currently recognized. Future research should employ high-throughput screening, targeted gene editing, and advanced cell-biological approaches to systematically identify peptide hormones involved in root hair formation and characterize their signaling pathways. Such efforts will be essential for constructing a comprehensive molecular framework to explain how peptide hormones coordinate root hair development with environmental adaptation.

## Peptide hormones in nodule development

**Figure 5:**
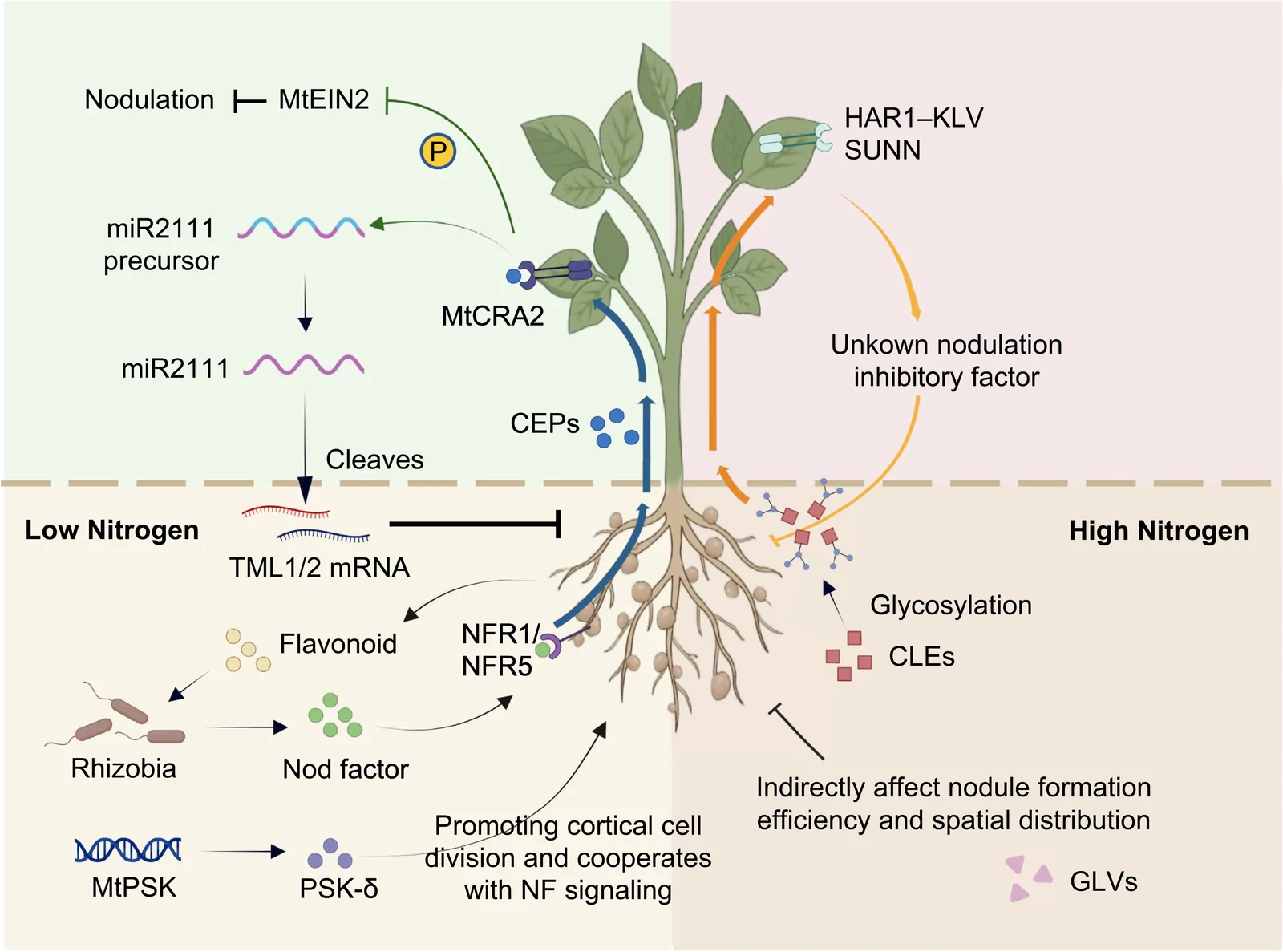
Modulation of nodule formation by peptide hormones. Peptide hormones modulate symbiotic nodule development and maintain nitrogen homeostasis. Under nitrogen-deficient conditions, root-derived CEPs mediate long-distance root-to-shoot-to-root communication, alleviating repression of nodule initiation. PSK-δ acts synergistically with Nod factor signaling to stimulate cortical cell division. In contrast, CLEs activate autoregulatory receptors in the shoot and trigger negative-feedback loops, restricting excessive nodule formation and carbon consumption. These peptide signals also respond to changes in phosphorus and iron availability, thereby adjusting the progression of nodulation. This sophisticated regulatory network dynamically coordinates resource allocation, fine-tunes nodule number, and maintains a balance between symbiotic nitrogen fixation and vegetative growth. CEP, C-terminally encoded peptide; CLE, clavata3/embryo surrounding region-related; PSK, phytosulfokine.

Nodules are specialized root-derived organs formed through symbiosis between legumes and rhizobia, enabling plants to fix atmospheric nitrogen and thereby reduce their reliance on soil nitrogen (Luo et al., 2024; Taleski et al., 2024). Nodule formation is a highly complex process involving precise host–microbe recognition, reciprocal signal exchange, and coordinated cell division and differentiation in both symbiotic partners (Roy et al., 2020; Xu and Wang, 2023). When legumes grow under nitrogen-deficient conditions, they secrete specific flavonoid compounds from their roots that attract compatible rhizobia (Subramanian et al., 2007). In response, rhizobia secrete lipochitooligosaccharide signaling molecules known as Nod factors (NFs), which are recognized by the plant cell-surface receptor nod factor receptor 1 (NFR1) and its co-receptor NFR5, thereby initiating the symbiotic signaling pathway (Long, 1996; Radutoiu et al., 2003; Peck et al., 2006; Yang et al., 2022). NFs are essential both for nodule organogenesis and for the establishment of rhizobial infection (Zhou et al., 2024) (Figure 5).

Rhizobial infection upregulates CK biosynthesis genes encoding isopentenyl transferases, leading to localized CK accumulation in the endodermis (van Zeijl et al., 2015; Mens et al., 2018). The resulting CK signal activates CRE1 receptor signaling, which induces expression of the master nodulation regulator *NIN* in cortical cells and promotes cell division (Liu et al., 2019; Tan et al., 2020). NIN further amplifies CK signaling through a positive feedback loop by activating *CRE1* (Vernié et al., 2015) and directly promotes nodule primordium initiation by inducing *NF-YA/B* and *LBD16/ASL18* (Roux et al., 2014; Soyano et al., 2019). The CK receptor LHK1 is essential for nodulation in both *Lotus japonicus* and *M. truncatula* (Murray et al., 2007; Tirichine et al., 2007), underscoring the role of CK as a critical early determinant of nodule development (Figure 5).

In addition to CK, multiple peptide hormones—notably CEPs and CLEs—play pivotal roles in orchestrating nodule initiation and development, as well as in providing feedback control over nitrogen homeostasis (Figure 5). By exerting either promotive or inhibitory effects on nodule formation, these signaling molecules constitute a sophisticated systemic regulatory network that integrates environmental nitrogen availability with endogenous developmental programs, thereby enabling precise and adaptive control of nodulation.

### Peptides that facilitate nodule development

Among the peptide hormones that promote nodulation, CEPs represent the most extensively studied family. During nodulation under low-nitrogen conditions, CK induces *MtCEP7* through the CRE1–NIN module, thereby initiating CEP-mediated nodulation signaling (Laffont et al., 2020). The resulting MtCEPs are translocated from roots to shoots, where they are perceived by the MtCRA2 receptor (Hermans et al., 2006; Tabata et al., 2014; Ohkubo et al., 2017). MtCRA2 not only promotes infection thread formation (Imin et al., 2013) but also phosphorylates ETHYLENE INSENSITIVE 2 (MtEIN2), thereby inhibiting its C-terminal cleavage and attenuating the inhibitory effect of ethylene on nodulation (Laffont et al., 2019; Zhu et al., 2020a). Moreover, MtCRA2 promotes miR2111 accumulation in both shoots and roots. In roots, miR2111 cleaves the transcripts of its target genes *too much love 1* and *2, thereby* relieving their repression of nodulation and promoting nodule formation (Mohd-Radzman et al., 2016; Gautrat et al., 2020). Thus, under nitrogen starvation, CEPs coordinate systemic shoot–root signaling networks that promote nodule formation and enhance the plant’s capacity to compensate for limited soil nitrogen availability (Figure 5).

Beyond their roles in nodulation, CEPs also contribute to arbuscular mycorrhizal symbiosis (AMS), primarily through their interaction with strigolactones (SLs). SLs are key regulators of shoot branching and root architecture, including the inhibition of LR development and promotion of root hair elongation. They also facilitate symbiotic interactions with arbuscular mycorrhizal fungi (AMFs) and rhizobia (Gomez-Roldan et al., 2008; Umehara et al., 2008; Wang et al., 2020; Sun et al., 2023). Under low-nitrogen conditions, root-derived CEPs in legumes such as *M. truncatula are transported to the shoots* (Imin et al., 2013), which subsequently generate a systemic feedback signal that travels to the roots. This signal upregulates key SL biosynthetic genes, increases SL accumulation, and thereby promotes the establishment of AMS (Muller et al., 2019; Karlo et al., 2020; Pedinotti et al., 2024). These findings suggest that peptide hormones may indirectly regulate root development and symbiotic interactions by modulating SL biosynthesis or signaling (Figure 5).

In addition to its potential role in root hair development, PSK promotes nodulation. Recent studies have identified a novel legume-specific peptide hormone, PSK-δ, that is generated through alternative splicing of MtPSK transcripts. PSK-δ promotes nodule organogenesis by stimulating cortical cell division and acts synergistically with the NF signaling pathway to facilitate nodule formation (Ochatt et al., 2018; Yu et al., 2022a) (Figure 5).

### Peptides that inhibit nodule development

Although nodules facilitate nitrogen fixation, their number must be tightly controlled because symbiotic nitrogen fixation requires a substantial investment of host-derived carbon; consequently, excessive nodulation can impose a considerable metabolic burden on the plant (Oono et al., 2011; Teyssendier de la Serve et al., 2026). When soil nitrogen is abundant, roots can acquire inorganic nitrogen directly from the soil, and the energetically costly nodulation process should therefore be limited (Lin et al., 2021). Plants must therefore coordinate the initiation and progression of symbiosis according to their nutritional status through a systemic process known as autoregulation of nodulation (Caetano-Anollés and Gresshoff, 1991). In this process, CLEs play a key role by restricting unnecessary nodule formation and preventing the excessive allocation of photosynthates to symbiotic tissues (Kosslak and Bohlool, 1984; Caetano-Anollés and Gresshoff, 1991; Yamada and Sawa, 2013). When rhizobia successfully infect roots and early nodule development is initiated, or when plants are exposed to high-nitrogen conditions, specific *CLE* genes are induced in roots. Examples include *LjCLE-RS1* and *LjCLE-RS2* in *L. japonicus* and *MtCLE12* and *MtCLE13* in *M. truncatula* (Okamoto et al., 2009; Miyazawa et al., 2010; Mortier et al., 2010). After glycosylation, the CLE peptides are transported through the xylem to the shoot, where they are perceived by shoot-localized receptors (Kassaw et al., 2017; Yoro et al., 2019). In *L. japonicus*, the receptor complex comprises HYPERNODULATION AND ABERRANT ROOT FORMATION 1 and KLAVIER (Krusell et al., 2002; Nishimura et al., 2002; Okamoto et al., 2013); in *M. truncatula*, the corresponding receptor is SUPER NUMERIC NODULES (Schnabel et al., 2005; Moreau et al., 2021) (Figure 5).

Once CLEs activate their receptors, shoot-derived inhibitory signals are generated and transported back to the roots through the phloem, where they suppress further nodule initiation and development. This negative feedback loop maintains nodule numbers within an optimal range, thereby preventing the excessive diversion of photosynthates to symbiotic tissues (Okamoto et al., 2013; Yamada and Sawa, 2013). CLEs and CEPs form a coordinated regulatory system that adjusts nodule number in response to soil nitrogen availability (Luo et al., 2024). Under low-nitrogen conditions, CEP signaling predominates and promotes nodulation. As nodules develop and nitrogen fixation progresses, or under high-nitrogen conditions, CLE signaling is activated to inhibit further nodulation, thereby optimizing resource allocation (Yamada and Sawa, 2013). Thus, the balance between CLE and CEP signaling plays an important role in fine-tuning nodule number according to the nitrogen status of the plant and its environment. Interestingly, homologs of *WOX5* are also expressed in nodules, and elevated expression has been detected in some hypernodulation mutants, suggesting that the conserved *Arabidopsis* CLV1/CLV2–WOX5 pathway that regulates root stem cells may also contribute to nodule meristem maintenance and termination (Osipova et al., 2012) (Figure 5).

In addition to their roles in LR development, GLVs also contribute to the inhibition of nodule formation. Five *GLV*/*RGF* genes are upregulated during nodule organogenesis in *M. truncatula*; overexpression of these genes or exogenous application of the corresponding synthetic peptides reduces nodule numbers by 25%–50% (Roy et al., 2024). Notably, GLV10 can influence LR and nodule positioning, shorten the lateral organ formation zone, and alter root morphology and the spatial pattern of nodule formation by increasing cortical cell number, repressing the expression of microtubule-associated genes, and modulating the expression of auxin-responsive genes (Whitford et al., 2012; Fernandez et al., 2020). These findings suggest that GLVs may not directly suppress rhizobial symbiosis but instead indirectly influence the efficiency and spatial distribution of nodule formation by remodeling RSA and modulating auxin signaling (Figure 5).

### Peptide-mediated adaptive regulation of nodule development under stress

Although root nodules enable legumes to acquire atmospheric nitrogen, their formation and function require substantial resources and are highly sensitive to abiotic stress. Under adverse conditions, legumes actively limit nodule formation to conserve resources and sustain growth and survival (Dhanushkodi et al., 2018; Isidra-Arellano et al., 2020; Zhu et al., 2025b; Yeremko et al., 2025). Recent studies have shown that certain peptide hormones play key roles in this response by activating local and systemic signaling pathways that restrict nodulation.

Under nutrient stress, particularly phosphate deficiency, *MtCLE33* expression is induced in M. truncatula to suppress nodule formation (Muller et al., 2019). This response highlights the coordinated regulation of nitrogen and phosphorus acquisition. In addition, IRON MAN peptides, which are rapidly and specifically induced in plant roots under iron-deficient conditions, influence nodulation through BRUTUS-mediated signaling by redirecting internal iron toward nodules and thereby supporting nodule function (Ito et al., 2024). However, whether peptide signaling contributes to the strong inhibition of nodule development imposed by other stresses, such as salinity, remains unknown. Determining whether salt-induced inhibition of nodulation involves inhibitory “nodule-brake” CLE peptides therefore represents an important direction for future research. Furthermore, novel inhibitory peptides involved in the repression of nodulation must be identified and characterized. Elucidating how these peptides restrict nodule formation under stress will provide insights into resource-allocation strategies under adverse conditions and provide valuable genetic targets for breeding crops with enhanced nodulation plasticity.

### Peptide hormones in root–microbe interactions

Beyond classic rhizobial symbiosis, peptide hormones also mediate plant interactions with AMFs and other rhizosphere microorganisms while coordinating RSA remodeling in response to multiple stresses.

CEPs act as nutrient-signaling hubs linking nitrogen and phosphorus availability, RSA plasticity, and AMF colonization. Under low Pi availability, MtCEP1 enhances arbuscular mycorrhizal symbiosis at fungal entry sites. This pathway integrates nitrogen and phosphorus status with adaptive root branching, thereby expanding the potential sites available for microbial colonization (Pedinotti et al., 2024; Taleski et al., 2024). In *Arabidopsis*, AtCEPs primarily regulate N-starvation responses and LR development, whereas in legumes, *MtCEP* genes have been co-opted to control both nodulation and mycorrhization, illustrating lineage-specific functional diversification (Tang et al., 2022; Taleski et al., 2024).

CLEs have dual roles in the autoregulation of mycorrhizal symbiosis and drought-induced root hair elongation. Under high Pi availability, MtCLE33/53 inhibit AMS through the SUPER NUMERIC NODULES receptor (Frei Dit Frey and Spallek, 2026), whereas tomato SlCLE10/11 exert similar inhibitory effects through SlCLV2 (Wulf et al., 2024; Frei Dit Frey and Spallek, 2026). Conversely, MtCLE16 promotes AMS via the CRN receptor (Bashyal et al., 2025). Notably, AMFs secrete CLE-like mimics, such as RiCLE1, to modulate host development (Le Marquer et al., 2019). Under drought stress, SlCLE10 activates SlMAPK6, which stabilizes ACC synthase2 (SlACS2) and enhances ethylene biosynthesis, thereby promoting root hair elongation and increasing root–soil contact (Wang et al., 2026).

Additionally, the PSK–PSKR1 module attenuates salicylic acid-mediated defense responses in the rhizosphere, thereby facilitating persistent colonization by *Pseudomonas* (Song et al., 2023). In contrast, the RALF–FER module induces ROS bursts and elevations in cytosolic Ca^2+^ levels, thereby restricting fungal pathogens (He et al., 2023).

Collectively, the CEP, CLE, PSK, and RALF families form a finely tuned regulatory network that balances symbiosis, defense, and RSA plasticity. While most studies have focused on single stresses, future research should determine how peptide-signaling networks integrate simultaneous climatic stressors, such as drought and heat, and how such integration influences plant–microbe interactions. Addressing this knowledge gap will be essential for improving crop resilience under global environmental change.

## Peptide hormones in root regeneration

Root regeneration is the process by which an injured root reconstructs a functional root tip through the coordinated reactivation of cell division and differentiation programs in the remaining tissues (Chen et al., 2024). This process depends on the rapid perception of wound signals, re-establishment of the root SCN, and recovery of meristem activity. Upon root tip damage, interconnected Ca^2+^ waves, ROS production, and peptide-mediated signaling activate the jasmonic acid pathway and rapidly induce expression of the master regulator ethylene response factor 115 (ERF115). ERF115-dependent transcriptional reprogramming subsequently promotes the re-entry of QC cells into the cell cycle. Antagonistic interactions between auxin and CK then re-establish tissue polarity and a new auxin maximum, ultimately enabling reconstruction of the root SCN and regeneration of the root tip (Ikeuchi et al., 2019; Xu et al., 2021; Garcia-Gomez and Ten Tusscher, 2024).

RGF1 plays a key role in root regeneration. Mutants lacking RGF1 or its receptors exhibit substantially impaired regenerative capacity, demonstrating the importance of this signaling pathway in rebuilding the stem cell system. Exogenous application of RGF1 can partially restore *PLT* expression and the function of the stem cell zone, thereby promoting SCN re-establishment in the *rgf1* mutant (Wang et al., 2025a). CLE peptides also contribute to the regeneration of stem cell-containing tissues. CLE41/TDIF promotes regeneration by maintaining and expanding the vascular SCN, thereby facilitating cambial reconstruction (Hirakawa et al., 2010).

As a key damage-responsive peptide hormone, RALF33 rapidly accumulates after root tip excision. It subsequently activates FER-dependent phosphorylation events that weaken the interaction between the transcriptional corepressor TOPLESS-related 4 and ERF115, thereby relieving the repression of ERF115 transcriptional activity. As a master regulator of root regeneration, ERF115 directly activates cell cycle-related genes, such as *CYCD6;1*, *CYCD3;3*, and *CDKB2;1*, driving QC cells to re-enter the cell cycle and initiate root regeneration (Shen et al., 2025). Although RALF33–FER signaling can activate ERF115, the enhanced regenerative capacity of *fer mutants indicates that this module also imposes a negative constraint on* regeneration, thereby maintaining tissue homeostasis (Shen et al., 2025). Single-cell transcriptomic analysis further suggests that the RALF33–FER module is highly active in less differentiated cell clusters, particularly those containing columella and QC cells, and that these cells exhibit greater regenerative potential in *fer mutants* (Shen et al., 2025). Together, these findings suggest that the RALF33–FER module prevents aberrant growth during regeneration by restricting excessive proliferation in specific cell populations and ensuring that regenerating cells differentiate in a spatially coordinated manner.

In addition to RALFs, another critical class of peptides regulating regeneration is represented by regeneration factor 1 (REF1), which has been identified in tomato and *Arabidopsis* as a local wound signal that functions independently of systemin (Yang et al., 2024b). When plant cells are damaged, REF1 is proteolytically cleaved and secreted, after which it activates its receptor kinase PORK1. Activated PORK1 induces the expression of the master transcription factor *SlWIND1* (a WIND1 homolog in tomato), thereby initiating cell reprogramming and promoting callus formation. Following root injury, rapid activation of this pathway reprograms cells adjacent to the wound into a pluripotent state, generating a cellular source for tissue regeneration. Moreover, REF1 can act synergistically with hormones such as auxin to drive callus formation and facilitate the precise reconstruction of the RAM and SCN (Ikeuchi et al., 2019). Notably, *SlWIND1* can also bind to the promoter of the *REF1* precursor gene, forming a positive feedback loop that amplifies REF1 signaling during the wound response.

In summary, peptide hormones function as central damage signals during plant root regeneration, and their associated regulatory networks provide an important framework for elucidating the molecular mechanisms of plant wound repair. Different peptide hormones precisely regulate discrete stages of root regeneration through distinct receptor-binding modes and downstream signaling pathways. The RALF33–FER module negatively regulates regenerative activity by modulating the balance between key transcription factors, whereas REF1–PORK1 signaling initiates cell reprogramming and promotes callus formation and root regeneration through a positive feedback loop. These two pathways exhibit distinct, cell-type-specific expression patterns and functional compartmentalization. Future investigations into the crosstalk among peptide signals and their integration with classical phytohormone pathways should help uncover fundamental regulatory principles governing plant regeneration. Such insights could open new avenues for enhancing stress resilience and accelerating crop breeding programs through targeted engineering of peptide signaling pathways.

## Concluding remarks and perspectives

Peptide hormones constitute a sophisticated “plant language,” mediating cell-to-cell communication to fine-tune RSA in response to developmental and environmental cues. This review systematically summarizes the “conductor” roles of peptide hormones in primary root growth, LR initiation, root hair formation, nodulation, and root regeneration. These signaling molecules not only directly regulate root cell division, differentiation, and elongation but also interact with classical phytohormones, ROS, and nutrient signals, thereby forming complex networks that coordinate root growth and developmental plasticity. Systematic studies of the identification and agronomic applications of plant-derived peptides have established strategies for discovering functional peptides in plant species and demonstrated their potential to promote crop growth, enhance nutrient-use efficiency, and improve stress tolerance. Collectively, these advances provide a valuable foundation for the agricultural application of peptide hormones (Yu et al., 2025b).

Understanding the functions of peptide hormones in RSA plasticity requires a spatiotemporal, whole-root-system perspective. RGF1 exemplifies the importance of such an integrative view. Early in root development, RGF1 acts in the QC and meristem to maintain PLT protein gradients and SCN integrity, thereby supporting primary root growth. During LR development, GLV6 and GLV10 fine-tune local auxin maxima within LRPs to prevent excessive or closely spaced LR formation and ensure optimal root branching patterns. Beyond these roles, RGF1 may influence root hair differentiation through ROS-dependent mechanisms and contributes to root regeneration after injury, whereas MtGLVs regulate legume nodulation through crosstalk with auxin signaling. How such spatiotemporal specificity is achieved at the receptor and transcriptional levels remains unclear. Future studies combining single-cell approaches with tissue-specific genetic perturbations should map these signaling dynamics along the root axis and across developmental stages.

Peptide hormones have been reported to enhance crop yield in some cases, potentially through the optimization of RSA. They therefore represent promising targets for crop improvement and the development of high-yielding germplasm, although their agronomic benefits require further validation under field conditions. For example, PSKs have been shown to promote primary root elongation and LR initiation in *Arabidopsis*, wheat, and pine trees under controlled conditions, suggesting that PSK signaling could contribute to improved nutrient uptake and yield (Wu et al., 2019; Geng et al., 2020). RGFs/GLVs can increase root-system surface area and enhance the acquisition of water, nitrogen, and phosphorus, effects that have been associated with increased yield in maize and rice (Fang et al., 2021b). Moreover, the balance between CEP and CLE signaling in legumes has significant potential for the precise regulation of nodulation and the optimization of plant nitrogen nutrition. Manipulation of this module may therefore provide new strategies for reducing nitrogen-fertilizer inputs while sustaining or improving crop productivity (Yamada and Sawa, 2013; Luo et al., 2024).

In addition to improving crop yield, peptide hormones represent promising targets for breeding stress-resistant germplasm. For example, RALFs not only regulate root development but also modulate plant immune responses to pathogens, making RALF signaling a potential target for improving crop resistance to soil-borne diseases (Abarca et al., 2021; Chen et al., 2025). Under abiotic stress, DVLs regulate ABA signaling to maintain root-tip stem cell activity during drought and saline–alkali stress, thereby enhancing plant survival and post-stress recovery (Yang et al., 2024a). Similarly, an extracellular peptide–receptor module identified in soybean significantly enhances disease resistance and may therefore provide a valuable target for the molecular breeding of disease-resistant crops (Yu et al., 2025a).

Several important caveats should nevertheless be considered. First, it remains unclear whether observed increases in yield can be attributed primarily to changes in RSA or whether they also involve other mechanisms, such as direct effects on shoot growth or carbon allocation. Second, almost all evidence has been obtained from controlled laboratory or greenhouse experiments involving limited genetic backgrounds; multi-environment field trials across different years, locations, and soil types are needed. Finally, systematic evaluations of genotype-by-environment interactions remain limited, and the performance of peptide-based strategies may vary considerably among cultivars and environments. Therefore, although peptide hormones hold considerable promise for crop improvement, their integration into breeding programs will require rigorous validation under diverse field conditions, together with further mechanistic dissection of their effects on plant growth and stress adaptation.

Beyond the development of improved crop germplasm, the exploitation of saline–alkali and drought-prone lands represents another important strategy for safeguarding food security. In this context, peptide hormones offer distinct advantages by regulating root adaptation to adverse environments. For example, IDA enhances root penetration through compacted or saline soils by promoting cell-wall relaxation and LR emergence (Kumpf et al., 2013; Zhu et al., 2019; Xu et al., 2020). CIFs strengthen Casparian strip formation in the endodermis, thereby restricting the entry of potentially toxic ions, including sodium, into the vascular system and significantly improving plant salt tolerance (Zhang et al., 2024a). Given their roles in regulating root gravitropism and spatial architecture, GLVs/RGFs may also serve as potential molecular targets for breeding crop varieties with modified RSAs, such as deeper or wider root systems, that are better suited to marginal lands (Jourquin et al., 2023; Xu et al., 2023). However, these possibilities must be validated in crop species and under field conditions before they can be translated into practical applications. In addition to conventional breeding strategies, engineered peptides may provide a complementary and tunable means of enhancing plant plasticity because of their high binding affinity, functional specificity, and ligand stability. The rational design of synthetic peptide variants could enable precise control over RSA and stress responses, thereby improving crop adaptability to complex and variable environments (Hu et al., 2025).

Although substantial progress has been made in peptide hormone research, systematic investigation is still required to elucidate the mechanisms underlying signaling-network integration, spatiotemporally specific regulation, and functional diversity across crop germplasm. To translate this knowledge into agricultural innovation, future efforts should prioritize the following strategic directions. (1) High-throughput screening and synthetic-biology design: employ genome-editing and synthetic-peptide-library technologies to identify or engineer peptide hormone variants that efficiently promote growth and enhance stress resistance and to construct “peptide hormone regulatory modules” for precision crop breeding. (2) Modeling peptide hormone–environment interaction networks: integrate artificial intelligence with systems biology to build dynamic regulatory models that predict the performance and efficacy of peptide hormones under field conditions. (3) Cross-species analysis of functional conservation and specificity: systematically characterize and validate peptide hormone functions in major food crops and economically important forest species and identify species-specific regulatory pathways that can provide molecular targets for breeding across different ecological zones. (4) Development of peptide hormone-based bioproducts: develop active peptide hormones and their agonists or antagonists as biostimulants for foliar or root application to enable more streamlined and precise agricultural management.

In summary, peptide hormones are key signaling molecules that integrate developmental cues and environmental signals to shape RSA. Achieving a comprehensive, multidisciplinary understanding of this sophisticated regulatory system—from its molecular mechanisms to its effects at the whole-plant level—is therefore crucial. Such knowledge will provide a strong foundation for crop genetic improvement and sustainable agricultural production, ultimately contributing to global food security.

## Funding

This work was supported by the 10.13039/501100001809National Natural Science Foundation of China (grant nos. 32430007, 32470326, and 32572460), the Taishan Scholar Foundation of Shandong Province (grant nos. tstp20231201 and tsqn202507001), the 10.13039/501100007129Natural Science Foundation of Shandong Province (grant no. ZR2024QC012), the 10.13039/501100014761Natural Science Foundation of Qingdao (grant no. 24-4-4-zrjj-132-jch), and the 10.13039/100009108Shandong University Future Scholars Talent Program.

## Acknowledgments

The authors declare no competing interests.

## Author contributions

Visualization, X.Z., H.T., G.L., and Z.Y.; Writing – original draft, X.Z., H.T., G.L., and Z.Y.; Writing – review and editing, Z.Y., Y.X., G.L., and Z.D.
